# The role of cation and anion structural modifications for the enhanced CO_2_ solubility of hydroxyl ammonium- and pyridinium-based ionic liquids

**DOI:** 10.1039/d6ra03083a

**Published:** 2026-05-26

**Authors:** Ahmed Mohamed Abdelmagid, Abobakr Khidir Ziyada, Abdalla Ahmed Elbashir, Zakaria A. Salih

**Affiliations:** a Department Chemical Engineering and Chemical Technology, Faculty of Engineering and Technology, University of Gezira Wad Medani 21113 Sudan bahooti@gmail.com; b General Studies Department, Jubail Industrial College Jubail Industrial City 31961 Saudi Arabia; c Department of Chemistry, College of Science, King Faisal University P. O. Box 400 Al-Ahsa 31982 Saudi Arabia aaeahmed@kfu.edu.sa; d Research and Training Station, King Faisal University P. O. Box 400 Al-Ahsa 31982 Saudi Arabia

## Abstract

This study investigates the CO_2_ capture efficiency of a series of ionic liquids (ILs) formed through the methodical modifications of cation and anion configurations. ILs based on tris-(2-hydroxyethyl) ammonium, containing acetate [Ac], butyrate [Bu], lactate [La], and ascorbate [As] anions, along with allyl [Ay]- and benzyl [Bz]-substituted ammonium and pyridinium chloride [Cl] salts, were synthesized and characterized using ^1^H and ^13^C NMR spectroscopies, elemental analysis, and Karl Fischer titration. Their CO_2_ solubility was investigated under pressures ranging from 1 to 20 bar and temperatures between 298.15 and 358.15 K. The solubility results indicated a distinct relationship with pressure: CO_2_ solubility rises as pressure increases for all ILs, while elevated temperatures diminish solubility and increase Henry's law constants. Among the anions examined, the [As]-based ILs had the greatest CO_2_ affinity, followed by [La]- and [Bu]-based ILs, suggesting that the anion structure and accessible free volume significantly affected gas absorption. Among the [Cl]-based ILs, [Bz]-based ILs exhibited superior CO_2_ solubility compared to their [Ay] counterparts, attributable to their increased van der Waals contacts and improved structural accommodation of CO_2_. The thermodynamic study utilizing Henry's law constants produced negative values for Δ*H*^0^ and Δ*S*^0^ and positive values for Δ*G*^0^ over the examined temperature range. The results demonstrate exothermic yet non-spontaneous dissolution under the examined circumstances. The values of Δ*H*^0^ and Δ*S*^0^ indicate that CO_2_ absorption is influenced by a balance of advantageous ion–gas interactions and solvent structuring effects. The results indicate that the targeted alteration of both cation and anion frameworks is a viable method for optimizing IL performance in CO_2_ capture applications.

## Abstract 2

This study investigates the CO_2_ capture efficiency of a series of ionic liquids (ILs) formed through the methodical modifications of cation and anion configurations.

## Graphical abstract

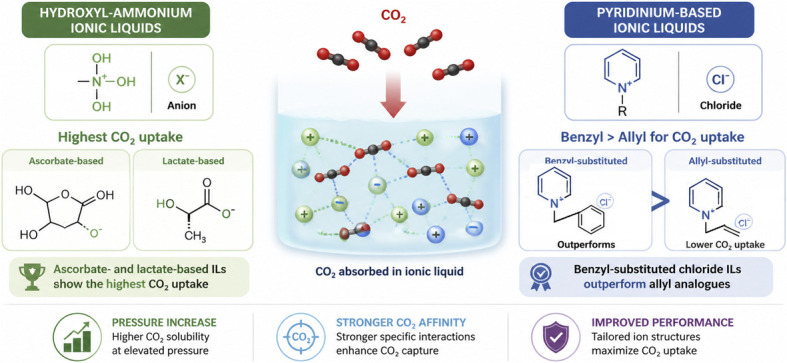

## 1. Introduction

The need for CO_2_ capture and separation technology has increased due to the fast industrialization of many nations.^1,2^ Effective CO_2_ capture and reducing the atmospheric CO_2_ levels will therefore continue to be crucial actions in the upcoming decades.^3,4^ Although natural carbon sinks like forests and oceans can assist in reducing the atmospheric CO_2_ levels, there is still a tremendous commercial and environmental demand for better carbon-capture materials.^5^ Aqueous amine-based solvents have long been used in industrial settings to absorb CO_2_, but these systems have a number of drawbacks, such as the high energy requirement for solvent renewal. Researchers have been driven to discover or create new materials that are better than common amines for CO_2_ separation due to political, economic, and societal considerations as well as the need for clean, sustainable technologies.

Ionic liquids (ILs) represent a promising option for CO_2_ capture, offering numerous opportunities to reassess and improve existing technologies and processes for CO_2_ capture.^6,7^ They have been extensively researched as a potential alternative to aqueous amine solutions due to their distinctive properties and their ability to enhance CO_2_ absorption capacity and selectivity through strategic design and the selection of suitable cation–anion combinations. The perceived value of ILs can be attributed to their significant characteristics, including substantial CO_2_ absorption capacity,^8,9^ elevated thermal stability, minimal volatility, nonflammability, extensive liquidus range, and solvation qualities.^10,11^

ILs often have melting points lower than 100 °C and are composed of inorganic and/or organic anions and cations.^2^ Several ILs of various types have been produced by mixing different anions and cations.^12^ ILs are a special kind of solvent that has several useful characteristics, such as being very effective at dissolving a variety of polar and nonpolar compounds, being very stable under both high and low temperatures, having very low vapor pressure, and having a large electrochemical window.

The classification of ionic liquids is typically based on the type of cation and anion they contain, rather than their fundamental cationic or anionic nature. There is a positively charged ion and a negatively charged ion in every ionic liquid, which means that every ionic liquid has both a cation and an anion.^13^ The key applications of cationic ionic liquids include catalysis,^13,14^ solvent substitution in synthesis, separation and extraction,^15^ electrochemical systems and batteries,^16^ and pharmaceutical and biomedical applications^17^ due to the significant influence of the cation structure on viscosity, polarity, stability, and biocompatibility.^17^ Anionic ionic liquids are particularly significant in fundamental catalysis,^16^ CO_2_ capture and gas separation,^18^ and functional materials,^16^ as the anion may profoundly affect hydrogen bonding, solvation, and ion-pairing dynamics.^16,17^

The industrial and technological uses of ILs are numerous, which include biomass processing, electroplating, and their use in solar cells, lubricants, and electrolytes, due to their desirable qualities.^19,20^ Although these solvents might not be considered very environmentally friendly when compared using the type 2 criterion, they are very eco-friendly when compared using the type 1 criterion (type 1 comparisons are process- and application-neutral, comparing solvents only on the basis of their mass, whereas type 2 comparisons are tailored to a specific application).^21^

Several researchers have reported gas solubility measurements, such as the solubility of CO_2_, for various ILs. For imidazolium-, ammonium-, and pyridinium-based ILs, although their gas solubilities are now being reported in the literature, there is still a need for data that aid in evaluating the potential of these ILs in various applications as well as elucidating their structural features that govern their solvating ability. At a specific temperature and pressure, a gas' solubility is the highest concentration that can be dissolved in a liquid. The gas molecules' rates of entry and exit into the solution are equal when the system is in equilibrium. A new equilibrium is achieved when the concentration of dissolved gas rises due to an increase in the number of gas molecules interacting with the surface of the liquid as a result of an increase in pressure.^22^ The solubility of a gas is determined by a combination of factors, including the polarizability of the gas, the dispersion forces, and the interactions between the gas and the solvent. The relative influence of each of these factors varies depending on the nature of the gas and the solvent concerned. It has been documented in numerous studies that the solubility of CO_2_ in ILs is predominantly determined by the strength of the interaction between CO_2_ and the anion.^23^

The primary disadvantages of utilizing various ILs in large-scale CO_2_ processes are their elevated cost and viscosity, which are deemed superior to those of conventional solvents.^24,25^ Considering that ILs are typically utilized in diluted forms with viscosities comparable to that of water, the challenge of high viscosity associated with ILs can be minimized.^26^ The low vapor pressure, low volatility, and excellent stability of ILs compared with those of existing solvents are essential properties that enhance their use for CO_2_ absorption. Because of their high volatility and high heat degradation, typical solvents used in the CO_2_ absorption process, such as monoethanolamine, have a substantial impact on both the efficiency and the cost of the process. The fact that ILs can be regenerated at 1 bar makes them a potentially cost-effective option for pre-combustion CO_2_ uptake. In addition to decreasing the cost of utilities involved, this would also minimize the gap in heat transfer between operations. The potential to renew ILs at 1 bar and elevated temperatures may reduce the cost of CO_2_ capture relative to the substantial expenses associated with conventional solvents, primarily due to the considerable equipment costs linked to vacuum regeneration processes.^26,27^ Prior studies evaluating the cost and performance of the IL process suggest that this technology is promising. Moreover, the customizable nature of ILs could be employed for the creation of cost-effective, thermally stable ILs with significant CO_2_ capture capacity and selectivity. Furthermore, the elevated degradation temperatures of ILs protect equipment from corrosion resulting from the reaction of ILs with contaminants.^28^ The tunable characteristics of ILs enable scientists to engineer them with features specifically suited for particular purposes.^2^

Computational studies have suggested that in the process of dissolving CO_2_ in ILs, the anion plays a more significant role than the cation.^29^ This is due to the fact that the anion is a stronger base, whereas CO_2_ is a Lewis acid. However, experimental data reveal that CO_2_ is more soluble in the [PF_6_] anion than in the [BF_4_] anion, implying that other factors, such as free volume, also play a role in CO_2_ dissolution.^25^ Potential for CO_2_ capture has been shown by functionalized ILs.^30^ Adding a reactive functional group to the cation or anion can greatly improve the solubility of CO_2_.^31^ In addition to fluoroalkyl groups, amine-functionalized ILs have a strong affinity for CO_2_ and are capable of capturing it by generating carbamates.^6,32^

The solubility of CO_2_ can be enhanced by incorporating oxygen-containing functional groups, such as ether and ester groups, into the cation and anion. This is achieved by utilizing the electron-deficient carbon atom's capacity to attract electronegative elements. Nevertheless, it is of the utmost importance to strike a compromise between the requirements for high CO_2_ solubility and the requirements for easy CO_2_ desorption from the IL.^33,34^ As a result, the large-scale commercial implementation of ILs for CO_2_ capture necessitates a thorough understanding of their physical and chemical characteristics. As a result, experimental procedures and data development are critical to the future of practical, cost-effective, and long-term CO_2_ capture utilizing ILs.

Even with the process of preparing ILs with functional groups, extensive research is required to ascertain the effect of cations with hydroxyl groups, allyl groups, or benzyl groups, as well as their combination with carboxylate- or chloride-based anions, on their CO_2_ capture efficiency.

In order to assess the effects of several structural variants on CO_2_ solubility, the current work carefully constructed IL structures. While the anions had a variety of structures and functional groups, the cations were paired with hydroxyl groups, allyl chains, benzyl groups, and different alkyl chains. The modified structures have characteristics that are known to increase a molecule's CO_2_-philicity, leading to increased CO_2_ solubility in ILs.

To investigate the solubility of CO_2_ in ILs, a comparison was made between the solubility of CO_2_ in ILs that had different combinations of cations and anions as well as ILs that were at the same pressure and different pressures. In order to assess the effects of several structural variants on CO_2_ solubility, the present work carefully designed IL structures. While the anions (acetate, butyrate, lactate, and ascorbate) had a variety of structures and functional groups, the cations were mixed with different alkyl chains and benzyl and allyl functionalities. The selected anions' (acetate, butyrate, lactate, and ascorbate) architectures had characteristics such as carboxylate, hydroxyl, carbonyl, alkyl chains and branched alkyl chains, which are known to improve CO_2_-philicity and result in the high solubility of CO_2_ in ILs.^35^ Additionally, the capacity, selectivity, and reversibility were determined by the distinct structural features of each anion family.^36–38^

ILs, which incorporated the cations tris-(2-hyroxyethyl) ammonium [HEA], allyl tris-(2-hydroxyethyl) ammonium [AyHEA], benzyl tris(2-hydroxyethyl) ammonium [BzHEA], allyl pyridinium [AyPy], and benzyl pyridinium [BzPy] and incorporated the anions acetate [Ac], lactate [La], butyrate [Bu], ascorbate [As], and chloride [Cl], were synthesized and reported in order to investigate the impact of pressure, cations, and anions on the solubility of CO_2_. Proton (^1^H) and carbon-13 (^13^C) NMR spectra, as well as CHNS elemental analysis (percentages of carbon (C), hydrogen (H), nitrogen (N), and sulfur (S)), were used to characterize the synthesized ILs. The effectiveness of these compounds in capturing CO_2_ was studied in the pressure range from 1 to 20 bar.

## 2. Materials and methods

### 2.1. Materials

Analytical-grade compounds were used for the synthesis of the presented ILs. The CAS numbers, sources, and grades of the utilized chemicals are as follows: acetic acid (64-19-7, Merck, 99%), allyl chloride (107-05-1, Fluka, 98), ascorbic acid (50-81-7, Sigma-Aldrich, 99%), benzyl chloride (100-44-7, Sigma-Aldrich, 99), butyric acid (107-92-6, Alfa Aesar, 99), ethyl acetate (141-78-6, Aldrich, 99.5), lactic acid (50-21-5, Fischer, 88.5), pyridine (110-86-1, Lab-scan, 99.5), and tris-2-hydroxyethyl ammonium (102-71-6, Avonchem, 97). All compounds were used without further purification.

### 2.2. Synthesis of ionic liquids

#### 2.2.1 Synthesis of tris-(2-hydroxyethyl) ammonium-based ILs

**Fig. 1:**
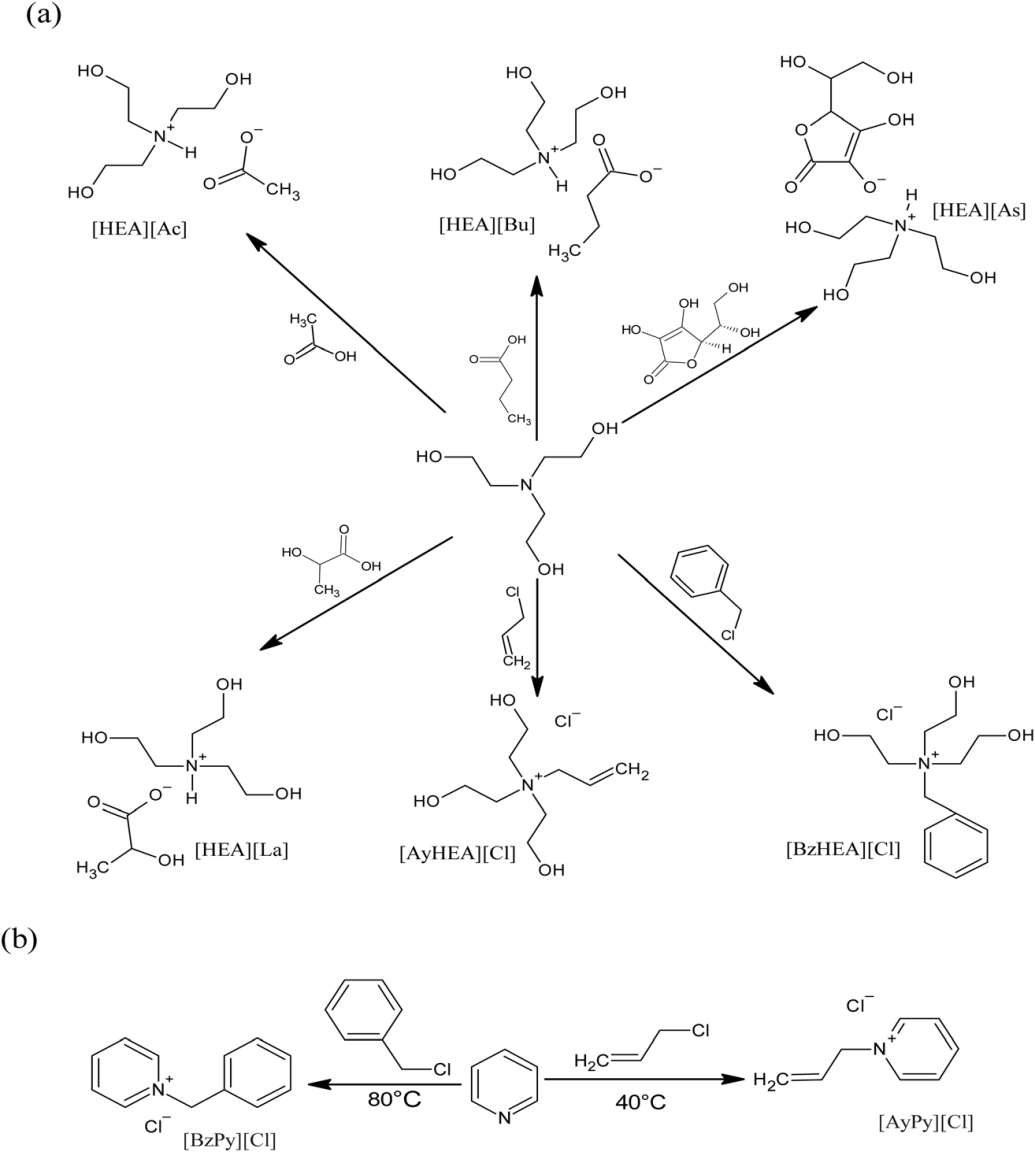
Synthesis route of ammonium-based ILs by acid/base neutralization.

A reflux condenser, a dropping funnel, a nitrogen gas source, and a magnetic stirring bar were fitted to a three-neck 500 mL flask. To ensure that the flask was free of moisture, first, a vacuum was applied; then, the surface of the flask was heated using a heat gun while the vacuum was being applied; finally, nitrogen gas was flushed through the flask. The flask was maintained under a dry nitrogen gas environment continuously. Equimolar amounts of tri-(2-hydroxyethyl) amine and acetic acid were used. The flask was placed in an ice bath while the acetic acid was added dropwise. The mixture was stirred vigorously for 12 hours at a temperature of 70–75 °C. The product was agitated for a minimum of 12 hours using a molecular sieve to decrease the water content and subsequently filtered to eliminate the molecular sieve residues. Finally, the product was dried for 48 hours in a vacuum oven to obtain the tris-(2-hydroxyethyl) ammonium acetate IL, [HEA][Ac].^39,40^ A diagram of the step of synthesis is presented in Fig. 1a.

The synthesis of tris(2-hydroxyethyl)ammonium butyrate [HEA][Bu], tris(2 hydroxyethyl)ammonium lactate [HEA][La], and tris(2-hydroxyethyl)ammonium ascorbate [HEA][As] was carried out in a manner that was similar to the above-given procedure, with acetic acid substituted with equivalent moles of butyric acid, lactic acid, and ascorbic acid, respectively.^40^ The synthesis route is shown in Fig. 1a.

#### 2.2.2 Synthesis of pyridinium-based ILs

The allyl pyridinium chloride IL, [AyPy][Cl] IL, was prepared by mixing 0.20 mol of allyl chloride and 0.19 mol of pyridine, both of which were dissolved in 20 mL of ethyl acetate. Subsequently, the mixture was stirred and heated at a temperature of 45 °C for 72 hours in a round-bottom flask kept on a hot plate, fitted with a reflux condenser, and connected to an inert gas to prevent air from entering the system. In order to remove the solvent and generate allyl pyridinium chloride, [AyPy][Cl], the product was dried in vacuum at a temperature of 80 °C. The synthesis route is shown in Fig. 1b.

The synthesis of benzyl pyridinium chloride, [BzPy][Cl], was achieved by repeating a technique that was quite similar to the one described above. However, this time, the allyl chloride was substituted with equal moles of benzyl chloride. Additionally, the mixture was heated for a period of 72 hours at a temperature of 75 °C. The synthesis route is shown in Fig. 1b.

#### 2.2.3 Synthesis of tris-2-hydroxyethyl ammonium chloride-based ILs

A round-bottomed flask equipped with a heating oil bath, a nitrogen inlet adapter, magnetic stirrer, and reflux condenser is flushed with dry nitrogen. Equimolar amounts of tris-(2-hydroxyethyl) amine and allyl chloride were dissolved in toluene. The reaction mixture was heated at 45 °C for 48 hours. The product was cooled to room temperature and washed with ethanol then, the remaining solvent was removed at 80 °C *in vacuo*. The product was dried in a vacuum oven for 72 hours to afford allyl tris-(2-hydroxyethyl) ammonium chloride [AyHEA][Cl]. A similar procedure used to synthesize benzyl tris-2-hydroxyethyl ammonium chloride [BzHEA][Cl] was followed to prepare chloride [AyHEA][Cl] by replacing allyl chloride with benzyl chloride.

### 2.3. Characterization

A nuclear magnetic resonance (NMR) magnetic spectrophotometer, Agilent 600 MHz Premium COMPACT, was used in order to obtain the ^1^H NMR spectra of the produced ILs. The solvent used was dimethyl sulfoxide (DMSO). The CHNS-932 elemental analyzer manufactured by LECO instruments was utilized in order to determine the percentages of carbon, hydrogen, nitrogen, and sulfur present in the prepared ILs. The instrument was standardized prior to each measurement by utilizing a standard calibration sample with a chemical composition that was determined by the supplier. The water content of the presented ILs was determined by a coulometric Karl Fischer titrator, DL 39 (Mettler Toledo), in conjunction with the CombiCoulomat fritless Karl Fischer reagent (Merck) and the Hydranal coulomat AG reagent (Riedel-de Haen). The results are given as the average of three separate measurements.^40,41^

### 2.4. Solubility of CO_2_ in ILs

The gravimetric technique was used to identify the solubility of CO_2_ in the prepared ILs utilizing a magnetic suspension balance (MSB). This approach is widely used to assess the solubility of gases in solids by measuring the weight change of the sample after absorption; however, it is less often utilized for liquids due to the potential influence of evaporation on the final weight of the sample. Due to the non-volatile features of the ILs, the gravimetric technique was used to determine their gas solubility.^42^

The magnetic suspension balance (MSB) is a multi-functional and accurate instrument for determining the solubility of gases in liquids. It allows real-time observation of mass changes, which is crucial for identifying equilibrium. Achieving equilibrium is essential prior to performing precise solubility measurements. The MSB may also verify the thorough degassing of the starting liquid, which is essential for precise findings.^42^ The magnetic suspension balance (MSB) from Rubotherm, Präzisionsmesstechnik GmbH, Bochum, Germany, was employed to ascertain the solubility of CO_2_ in the synthesized ILs. The MSB utilized a magnetic suspension coupling that included an electromagnet and a suspension magnet, with the electromagnet electronically regulated to sustain the suspension magnet in a condition of frictionless levitation. The microbalance had an accuracy of 0.001 mg and a repeatability of ±0.020 mg. The pressure and temperature in the measurement cell were regulated to within ±0.05 bar and ±0.2 °C, respectively, as documented in the literature.^43^

To obtain precise weight measurements, all potential environmental disturbances impacting the sample were reduced, controlled, and measured, with the exception of buoyancy, which was quantified and adjusted utilizing the MessPro software for instrument control and data recording. The MSB technique to measure the solubility of CO_2_ in the synthesized ILs involved four primary steps: blank measurement, sample dryness, buoyancy correction, and solubility measurements. An empty sample container was weighed, and its volume was quantified in the blank measurement. The sample was subsequently dried to eliminate any moisture and volatile compounds. The volume of the sample in the container was accurately obtained by a buoyancy measurement. The measurement method started at a stable temperature and progressively increased the pressure to the specified value under continuous monitoring. The solubility measurement was deemed complete when the MSB exhibited no more mass rise. The equipment, sample preparation, and measurement methodology are thoroughly explained.^43–45^

## 3. Results

### 3.1. Characterization

The structures of the prepared ILs were confirmed by NMR spectroscopy and elemental analysis (CHNS). The findings verified the predicted structures. The NMR spectra are presented in the SI (Fig. S1–S6) and reported with the elemental analysis results as follows:

#### 3.1.1 [HEA][Ac]

*δ*_H_ (600 MHz; DMSO): 4.74 (s, 1H, HO–CH–), 3.46 (s, 1H, N–CH–CH), 2.70 (q, R_2_N–CH), 1.78 (m, R–H). Elemental analysis: % actual C, 46.13; H, 8.91; N, 6.82; O, 38.14. C_8_H_19_NO_5_. % Theoretical C, 45.92; H, 9.15; N, 6.69; O, 38.23. Mass fraction of water: 171 × 10^−6^.

#### 3.1.2 [HEA][As]

*δ*_H_ (600 MHz; DMSO): 3.805 (q, ROH), 3.38 (m, R_2_N–CH), 2.52 (m, NC–CH), 2.05 (s, R–H). Elemental analysis: % actual C, 44.44; H, 6.84; N, 4.32; O, 44.4. C_12_H_23_NO_9_. % Theoretical C, 44.30; H, 7.13; N, 4.31; O, 44.26. Mass fraction of water: 128 × 10^−6^.

#### 3.1.3 [HEA][Bu]

*δ*_H_ (600 MHz; DMSO): 3.98 (d, HO–CH), 3.39 (m, ROH), 2.86 (s, R_2_N–CH), 2.53 (q, NC–CH), 2.11 (q, RCOO), 1.4 (m, R_2_NH), 0.83 (q, RH). Elemental analysis: % actual C, 50.83; H, 9.38; N, 5.93; O, 33.86. C_10_H_23_NO_5_. % Theoretical C, 50.62; H, 9.77; N, 5.90; O, 33.71. Mass fraction of water: 159 × 10^−6^.

#### 3.1.4 [HEA][La]

*δ*_H_ (600 MHz; DMSO): 5.32 (s, RCOOCH_3_), 3.66 (m, ROH), 3.1 (q, R_2_NH), 2.03 (s, R_2_N–CH), 1.1 (m, R–H). Elemental analysis: % actual C, 45.56; H, 8.07; N, 5.91; O, 40.46. C_9_H_21_NO_6_. % Theoretical C, 45.18; H, 8.85; N, 5.85; O, 40.12. Mass fraction of water: 178 × 10^−6^.

#### 3.1.5 [AyHEA][Cl]

*δ*_H_ (600 MHz; DMSO): 6.06 (RCONH), 5.6 (RCOO^−^), 4.454 (s, R_2_HC=CHR_2_), 3.8 (ROH), 3.45 (R_2_NCH), 2.5 (R_2_NH). FTIR-ATR *υ*_max_/cm^−1^: 3356 (NH), 3561 (OH), and 3060 (–HC=CH). C_9_H_20_ClNO_3_. Elemental analysis: % actual C, 47.89; H, 8.93; N, 6.21; O, 21.27. C_9_H_20_ClNO_3_. % Theoretical C, 47.89; H, 8.93; N, 6.21; O, 21.27. Mass fraction of water: 265 × 10^−6^.

#### 3.1.6 [BzHEA][Cl]

*δ*_H_ (600 MHz; DMSO): 7.46 (m, aromatics), 4.3 (ROH), 3.97 (R_2_N–CH). Elemental analysis: % actual C, 56.62; H, 8.04; N, 5.08; O, 17.41. C_13_H_22_ClNO_3_. % Theoretical C, 56.62; H, 8.04; N, 5.08; O, 17.41. Mass fraction of water: 242 × 10^−6^.

#### 3.1.7 [AyPy][Cl]

*δ*_H_ (600 MHz; DMSO): 9.06 (m, H–C*C–H), 9.06 (t, H–C*N*C–H), 8.24 (d, H–C*CH*C–H), 8.24 (m, HC*CH), 8.74 (s, H–C*C–H), 5.70 (m, H–CH–C(sp^2^)–H), 5.70 (m, HC = CH_2_), 5.07 (m, H–C(sp^2^)–H), 5.03 (m, H–C(sp^2^)–H). Elemental analysis: % actual C, 61.45; H, 6.76; N, 9.5; Cl, 22.29. C_8_H_10_ClN. % Theoretical C, 61.74; H, 6.48; Cl, 22.78; N, 9.0. Mass fraction of water: 285 × 10^−6^.

#### 3.1.8 [BzPy][Cl]

*δ*_H_ (600 MHz; DMSO): 9.34 (q, H–C*N*C–H), 8.64 (m, H–C*C–H), 8.19 (q, H–C*CH*C–H), 7.60 (t, H–C*C–H), 7.44 (q, H–C*CH*C–H). Elemental analysis: % actual C, 70.06; H, 5.87; N, 6.97; Cl, 17.10. C_12_H_12_ClN. % Theoretical C, 70.07; H, 5.88; Cl, 17.24; N, 6.81. Mass fraction of water: 261 × 10^−6^.

### 3.2. Solubility of CO_2_ in ILs

In this work, we compared the affinity of several ILs with the same cation and different anions and also with the same anion and different cations for CO_2_ capture. The impact of these anions and cations on CO_2_ solubility was investigated using the [HEA] cation with four different anions ([Ac], [Bu], [La], and [As]) and two ammonium-based and two pyridinium-based cations linked with allyl and benzyl groups ([AyHEA], [BzHEA], [AyPy], and [BzPy]) incorporating the same anion [Cl].

Table 1 presents the mole fraction as a function of pressure for the experimental CO_2_ solubility measurements performed for the synthesized ILs at a temperature of 298.15 K and pressures of 1, 5, 10, 15, and 20 bar.

**Table 1:** CO_2_ solubility in the prepared ILs at 1, 5, 10, 15, and 20 bar as a mole fraction of CO_2_ (*x*_CO_2__)^a^

Pressure (bar)	CO_2_ mole fraction				
ILs	1	5	10	15	20
[HEA][Ac]	0.026	0.138	0.244	0.340	0.406
[HEA][As]	0.062	0.218	0.385	0.501	0.588
[HEA][Bu]	0.040	0.176	0.317	0.429	0.545
[HEA][La]	0.053	0.198	0.339	0.456	0.564

a Standard uncertainties, *u*, of temperature, pressure and MSB (mass reading) are 0.05 K, 0.25 kPa and 0.00002 g, respectively. The combined standard uncertainties of the CO_2_ solubility in the [HEA][Ac], [HEA][As], [HEA][Bu], and [HEA][La] ILs are 0.0087, 0.070, 0.0082, 0.0089 and 0.0123, respectively.

**Fig. 2:**
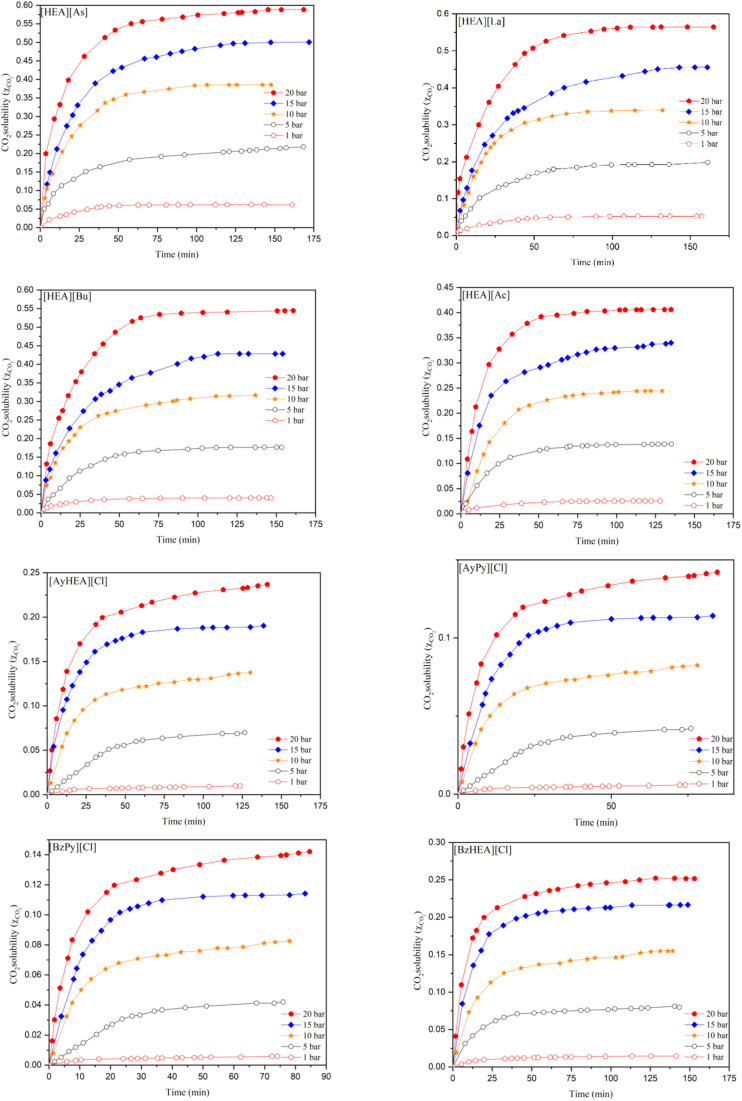
Effect of time on CO_2_ solubility in [HEA][Ac], [HEA][As], [HEA][Bu], [HEA][La], [BzHEA][Cl], [AyHEA][Cl], [BzPy][Cl], and [AyPy][Cl] at the pressures of 1, 5, 10, 15, and 20 bar.

The CO_2_ solubility in the [HEA] cation incorporating [Ac], [Bu], [La], and [As] anions at a temperature of 298.15 K and pressures of 1, 5, 10, 15 and 20 bar was measured, and the time-dependence of CO_2_ uptake by the studied ILs is reported in Tables S1–S8 in the SI and is shown in Fig. 2 in terms of the mole fraction (*x*_CO_2__) *versus* time.

The solubility results in Fig. 2 reveal that the solubility of CO_2_ in all studied ILs is positively correlated with an increase in pressure. This effect may be ascribed to the rise in pressure, which helps gas molecules dissolve in the ionic liquid solution in order to minimize the added pressure as much as possible. Recent data indicate that CO_2_ solubility in ILs is affected by factors beyond conventional interaction strength and free volume. Seki *et al.*^46^ proved that interactions alone cannot entirely elucidate CO_2_ sorption in ILs, contrary to prior assumptions, and that the robust Lewis acid–base interactions between ILs and dissolved CO_2_ are not the only determinants of CO_2_ solubility.

The results show that [HEA][Ac] reaches equilibrium in a shorter time than the other anions. This might be due to the lower viscosity of this anion.^40^ Gas diffusion in an IL depends on the viscosity of the IL; as the viscosity increases, the diffusion over time becomes greater. This increases the time required to reach equilibrium.^42^ The time required by the six ILs to reach equilibrium is less than that reported for the ILs incorporating the amine functionality (180 min)^42^ and is also not as long as that reported for non-functionalized imidazolium-based ILs (90–180 min).^47,48^ However, it is far less than the time required by [Emim][NTf_2_] (1800–2400 min) and [Bmim][NTf_2_] (2160–2880 min).^42^ The solubility of CO_2_ increases with increasing pressure for all six ILs, as expected, as increased pressure will force the gas into the IL.^49^

Fig. 2 demonstrates that the capture process is most efficient at the beginning of the process and that performance is significantly influenced by the IL structure, time, and pressure. These findings indicate an almost-linear increase in CO_2_ solubility with pressure for each IL, exhibiting significant pressure-solubility correlations across the 1–20 bar range. Among the studied ILs, CO_2_ capture at 20 bar is around 9.5–23.7 times greater than that at 1 bar, with the highest capacity exhibited by the [HEA]-based ILs.

The Pearson correlation (*r*) was used to analyze the relationship between pressure and solubility values. CO_2_ solubility exhibits a significant and almost-linear rise with pressure across all the studied ILs, with pressure-solubility correlations remaining consistently high for each IL (*r* = 0.988–0.996). The highest capture capacities are recorded for the [HEA]-based ILs, yielding 0.588–0.564 mole fraction at 20 bar, in contrast to 0.142–0.151 for pyridinium chlorides. The pressure impact is most pronounced for the [HEA]-based ILs, where solubility at 20 bar is 9.5–15.6 times more than that at 1 bar, whereas the pyridinium-based ILs exhibit comparable but much lower absolute absorption. The obtained results reveal that both IL cation and anion chemistries significantly influence CO_2_ affinity, with the [HEA] family affording the most suitable combination of enhanced capacity and significant pressure response.

Nonlinear regression of the time-dependent uptake curves using a first-order saturation model (*q*_*t*_ = *q*_*e*_(1 − *e*^−*kt*^), where *q*_*t*_, *q*_*e*_, *k*, and *t* are the amount adsorbed at time *t*, the equilibrium capacity, the first-order rate constant, and time, respectively) provides reliable estimates of the equilibrium capacity and capture rate for each IL.^50^ The first-order saturation model-fitted parameters (nonlinear fits) reported in the SI (Table S11) reveal a clear variation in the capacity and kinetics between the ILs, which is in accordance with the Pearson correlation analysis. The calculated rate constants (*k*) imply that uptake is efficient in all ILs; nevertheless, the most rapid equilibrium reached does not consistently correspond with its highest capacity. [BzPy][Cl] exhibits a comparatively high rate constant but a low equilibrium capacity, *q*_*e*_, indicating that it attains a low plateau rapidly, whereas [HEA][As] integrates a substantial capacity with a comparatively high uptake rate. This pattern is advantageous for process design because it differentiates between ILs that are simply quick and those that are both quick and high-capacity. In addition, the kinetic model's suitability for explaining the measured data is supported by the small confidence intervals, as shown in Table S11.

#### 3.2.1 Impact of anions on CO_2_ solubility

The theory of the anion effect speculates that the anion has a stronger effect on the CO_2_ solubility in ILs than the cation.^51^ The absorption of CO_2_ is affected by the organization of ions in the ILs, direct CO_2_–ion interactions, and the ratio of unoccupied space in pure ILs. This preformed unoccupied space is regularly dispersed throughout the IL and needs to be expanded by slight ion displacements to accommodate CO_2_. The amount of preformed unoccupied space is a good indicator of ion cohesion in ILs. Weak electrostatic cation–anion interaction densities in ILs, *i.e.*, weak ion cohesion, lead to larger average distances between ions and hence more unoccupied space.^52^ Weak ion cohesion facilitates ion displacement to enable the expansion of empty space to accommodate CO_2_. Moreover, it has been demonstrated that the ion density, which in turn is determined by the ion size, primarily determines the strength of ion cohesion. Additionally, ion cohesion is influenced by the local electrostatic interactions among ion moieties between which CO_2_ is inserted and which do not depend on the ion density.

**Fig. 3:**
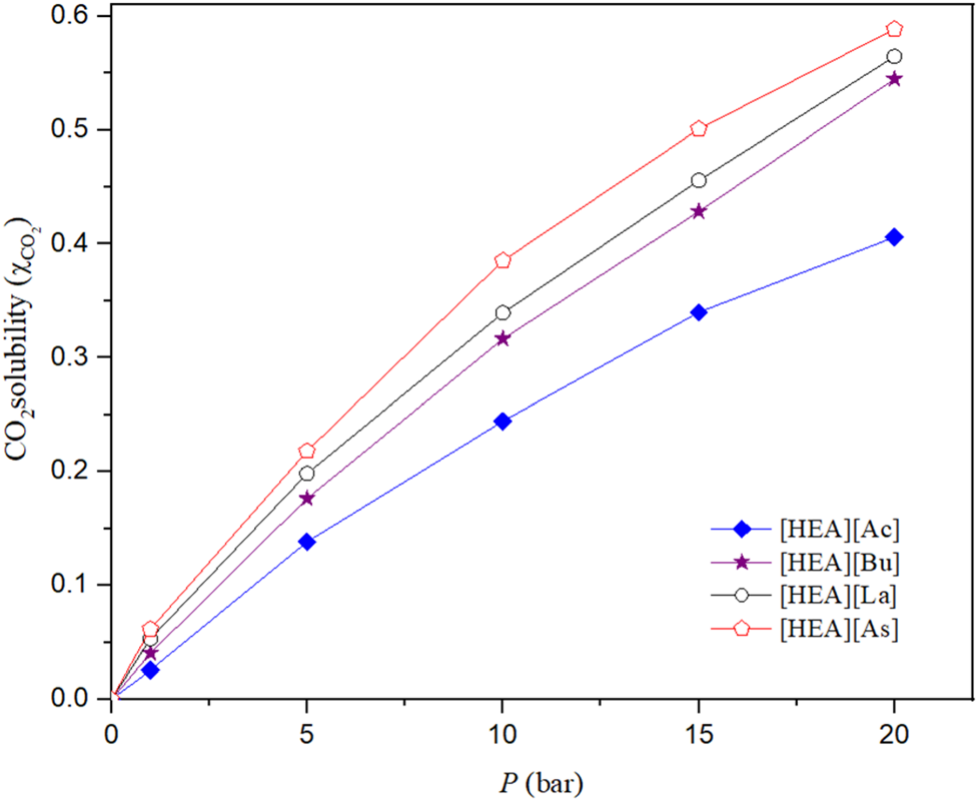
Effect of [Ac], [Bu], [La], and [As] anion-based ILs on CO_2_ solubility at 298.15 K.

The CO_2_ solubilities exhibit a linear relationship with pressure but exhibit a nonlinear trend as pressure increases for all the studied ILs. Fig. 3 shows the CO_2_ solubilities of the [HEA][Ac], [HEA][As], [HEA][Bu], and [HEA][La] ILs. Further, the solubility is clearly dependent on the choice of the anion, as shown in Table 1, which, by experimental proof, is in agreement with the reported literature.^53^

**Fig. 4:**
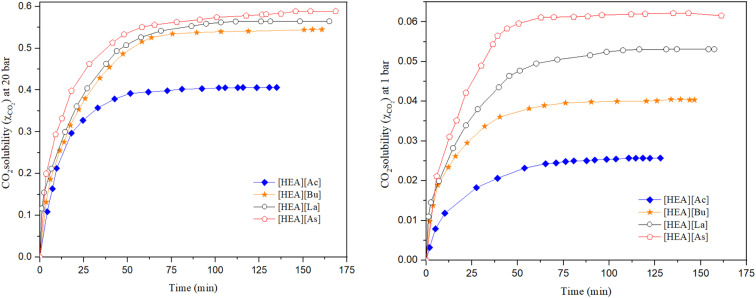
Effect of anion on CO_2_ solubility in [HEA][Ac], [HEA][As], [HEA][Bu], and [HEA][La] at the pressures of 1 and 20 bar.

The prepared ILs were used to investigate the influence of varying anion structures on the ability of each IL to absorb CO_2_. The CO_2_ solubility in the [HEA] cation incorporating [Ac], [Bu], [La], and [As] anions at a temperature of 298.15 K and pressures of 1, 5, 10, 15 and 20 bar was measured, and the solubility of CO_2_ in the studied ILs is reported in Table 1 in terms of the mole fraction (*x*_CO_2__). The results show that CO_2_ solubility is dependent on the choice of the anion, which agrees with the reported literature. Overall, the factors that determine the solubility of CO_2_ in ILs have been identified consistently across a large variety of constituting ions through molecular dynamics simulations.^54^ The isotherms showing the effect of the anion on the solubility of CO_2_ in ammonium-based ILs at 298.15 K are shown in Fig. 3 and 4, and the corresponding solubilities are presented in Table 1.

The IL with the [As] anion exhibits a considerably higher affinity for CO_2_ than the ammonium-based ILs incorporating [La], [Bu], and [Ac] anions and the same cation. The [As] anion has several features that are known to enhance a molecule's CO_2_-philicity and lead to the good solubility of CO_2_, such as carboxyl, hydroxyl, and double bond functionalities, long alkyl chains and branched alkyl chains.^52^ In addition, another major factor that affects the capacity for CO_2_ solubility is the molar free volume of the IL. In general, the larger the molecular size of the anion, the larger the free volume that CO_2_ can occupy.^55^ The molar free volume of the ionic liquid has a more significant impact than the basicity of the anion, which is an important factor in determining CO_2_ solubility in ILs. Furthermore, the anion exhibits minimal toxicity and is unlikely to exhibit the environmental persistence of fluorinated ILs while demonstrating a substantial capacity for CO_2_ solubility.

The comparatively enhanced solubility of CO_2_ in the [La]-based IL, compared to the [Bu]- and [Ac]-based ILs, may result from greater interactions between CO_2_ and the hydroxyl and branched methyl substituents on the [La] anion.^35^ There are two recognized forms of interactions: the acid–base interaction between CO_2_ and the anion in ILs and the interaction between CO_2_ and the methyl group in the anion.^23^ Actually, the solubility of CO_2_ increases with increasing number of hydroxyl groups in the anion.^23^ Compounds containing hydroxyl groups exhibit exceptional stability, imparting several advantageous features to them.^6^ Furthermore, the comparatively higher solubility of CO_2_ in [Bu]-based ILs, compared with [Ac]-based ILs, is attributable to the longer alkyl chain of the [Bu] anion, which enhances van der Waals-type interactions between the gas and the liquid.

The strength of the interactions cannot be solely responsible for the solubility of CO_2_ in ILs, as reported in the literature,^46,56,57^ and also the free volume. In addition, the ambient-pressure molar volumes increase with increasing chain length; this effect diminishes at higher amounts of dissolved CO_2_, and the molar volumes of all ILs become very similar.^58^ At these pressures, the IL consists of more than 70% mole of CO_2_, and thus, the properties are better correlated with CO_2_ than the ambient-pressure IL properties. The solubility of CO_2_ in the presented ILs increases rapidly to 0.3–0.4 mole fraction (CO_2_/IL) for pressures up to 10 bar, but at higher pressures, the increasing rate slows down, and the solubility finally levels off. The fast solubility increase in the low-pressure range may be due to Henry's sorption in the inter-ion space.

The presented results are in good agreement with those reported by Yuan *et al.* (2007) for the CO_2_ solubility in eight different hydroxyl ammonium ILs, including 2-hydroxyethylammonium incorporating formate, acetate, and lactate, at temperatures ranging from 303 to 323 K and pressures reaching 10.98 MPa.^1^ They confirm that hydroxyl-functionalized ammonium cations with carboxylate anions may accomplish competitive CO_2_ uptake among physisorbing ILs because their [HEA][acetate] findings at 303 K are largely similar to the values obtained in the current investigation.^53^ The investigated CO_2_ solubility in trimethylbutylammonium- and imidazolium-based ILs incorporating the [Tf_2_N] anion shows a CO_2_ mole-fraction solubility on the order of 10^−2^, with values similar to each other across the studied cations when the Tf_2_N^−^ anion remains constant.^59^ This suggests that the [HEA][carboxylate] ILs in the present study exceed traditional ammonium-based ILs in CO_2_ absorption, especially considering their fluorine-free, cost-effective carboxylate anions.

#### 3.2.2 Impact of cations on CO_2_ solubility

The cations of ILs are normally bulkier than the corresponding anions and provide inter-ion spaces where CO_2_ can squeeze in. As the openings between the cation and anion of the IL are large, CO_2_ molecules can easily enter that space. However, these openings are continuously filled by CO_2_ molecules with increasing pressure. Eventually, the spaces are filled up, leaving no more room for CO_2_ molecules to get in. To allow more CO_2_ molecules to enter the IL, it is necessary to expand the inter-ion space. As this expansion requires energy, only small amounts of CO_2_ can penetrate the IL. This is why ILs show that particular leveling off solubility behavior. The influence of the IL cation on CO_2_ solubility in ILs was investigated by comparing the effect of different functional groups attached to the cation on the solubility of CO_2_ in these ILs.

**Fig. 5:**
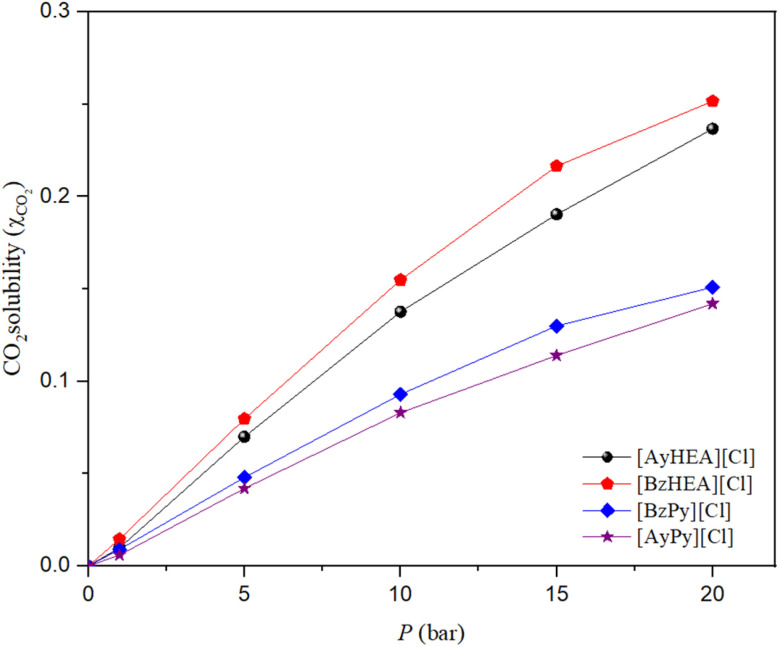
Effect of [HEA] and [Py] cation-based ILs on CO_2_ solubility at 298.15 K.

Allyl and benzyl functional groups may be easily incorporated into ammonium systems by the quaternization technique. They are often liquids, with some exhibiting lower viscosity than their saturated equivalents.^40^ The addition of a second functional group enhances the potential for interaction between the π-system in the benzyl and allyl groups and CO_2_. Furthermore, these ILs were studied to determine the influence of different functional groups on CO_2_ solubility in ILs. The solubility of CO_2_ in [BzHEA][Cl], [AyHEA][Cl], [BzPy][Cl], and [AyPy][Cl] at a temperature of 298.15 K and pressures of 1, 5, 10, 15 and 20 bar was measured, and the time-dependence of CO_2_ uptake by the studied ILs is reported in Tables S5–S8 in the SI and is shown in Fig. 5.

The mole fraction of CO_2_ absorbed by [BzHEA][Cl], [AyHEA][Cl], [BzPy][Cl], and [AyPy][Cl] at a temperature of 298.15 K is reported in Table 2. When comparing the ILs with the same anion, changing the cation can clearly change CO_2_ solubility, which might be responsible for the difference in CO_2_ solubility, as shown in Fig. 5. These findings are in line with the trends reported in the literature. However, ammonium-based ILs would provide higher CO_2_ solubility than pyridinium-based ILs due to their longer alkyl chain length, higher density and, thus, more free volume.^35,60^ There might be other factors that affect CO_2_ solubility, such as viscosity and polarity.^61^

**Table 2:** CO_2_ solubility in the prepared ILs at 1, 5, 10, 15, and 20 bar as a mole fraction of CO_2_ (*x*_CO_2__)^a^

Pressure (bar)	CO_2_ mole fraction				
ILs	1	5	10	15	20
[BzHEA][Cl]	0.015	0.079	0.155	0.217	0.252
[AyHEA][Cl]	0.010	0.070	0.138	0.190	0.237
[BzPy][Cl]	0.009	0.048	0.093	0.130	0.151
[AyPy][Cl]	0.006	0.042	0.083	0.114	0.142

a Standard uncertainties, *u*, of temperature, pressure and MSB (mass reading) are 0.05 K, 0.25 kPa and 0.00002 g, respectively. The combined standard uncertainties of the CO_2_ solubility for the [AyHEA][Cl], [BzHEA][Cl], [AyPy][Cl], and [BzPy][Cl] ILs are 0.0145, 0.0115, 0.0133, and 0.0123, respectively.

The cation incorporated with the benzyl group shows a higher capacity for CO_2_ solubility than that incorporated with the allyl group ([BzHEA][Cl] > [AyHEA][Cl] and [BzPy][Cl] > [AyPy][Cl]), which can be mainly attributed to the benzyl group's ability for enhancing the van der Waals-type interactions between the gas and the liquid more than the allyl group as a result of its greater number of π-electrons.

A recent study involving 19 types of ILs revealed that pyridinium [NTf_2_]-based ILs exhibit superior CO_2_ solubility within the [NTf_2_] anion group, with free volume being a critical factor (increased fractional free volume is associated with enhanced CO_2_ permeance and solubility across cation families).^62^ Moreover, another study reported that the Henry constant for CO_2_ solubility in six pyridinium-based ILs incorporating [Tf_2_N] at temperatures ranging from 298.15 to 333.15 K and pressures up to 28.98 bar is around 27 at 298 K, which is lower than that of the chloride-based pyridinium ILs reported in this research.^24^ This supports the interpretation that the inferior CO_2_ uptake of [BzPy][Cl] and [AyPy][Cl] relative to [HEA][carboxylate] ILs in the present study is primarily an anion effect—Cl^−^ provides less free volume and weaker CO_2_ interactions than carboxylate or sulfonylimide anions.^62^

Moreover, based on the experimental results, there is a relationship between the absorption of CO_2_ and the density and molecular volume of the ILs. As the density decreases and the molecular volume increases, the fractional free volume increases and, thus, the solubility of CO_2_ increases.^43,44^ At 1 to 20 bar and 298.15 K, there is only a marginal difference between the CO_2_ solubility in [BzHEA][Cl] and [BzPy][Cl] and that in [AyHEA][Cl] and [AyPy][Cl], which is mainly attributed to two contradictive factors: [BzHEA][Cl] and [BzPy][Cl] have a higher free volume than [AyHEA][Cl] and [AyPy][Cl], and [BzHEA][Cl] and [BzPy][Cl] have a higher density than [AyHEA][Cl] and [AyPy][Cl]. A strong linear correlation is observed between CO_2_ solubility and free volume.^8^ The [BzHEA] cation contains a benzyl group and has a higher molar mass than [AyHEA]. This creates a more open and less densely packed IL structure, providing additional space for CO_2_ molecules to be accommodated compared to [AyHEA].

Furthermore, at standard pressure and temperature, conventional imidazolium ILs containing [Tf_2_N], [PF_6_], and [BF_4_] anions attain a CO_2_ solubility of 3.3 mol% (Henry's constants ranging from 30 to 50), reflecting the practical difficulties associated with low-pressure CO_2_ capture from flue gas emissions.^2^ Conversely, the [HEA][carboxylate] ILs in the present investigation attain *x*_CO_2__ = 0.026–0.062 at only 1 bar—above this threshold—using non-fluorinated, biodegradable anions.

#### 3.2.3 Henry's constant

The gas solubility in a liquid is often expressed using the Henry's law constant. The Henry's law constant correlates the equilibrium mole fraction of a material in the liquid phase with its partial pressure in the gas phase.^63^ For all typical ILs, gas solubility diminishes with rising temperature and decreasing pressure. Under ideal conditions, gas solubility may be expressed as a Henry's law constant. At equilibrium and infinite dilution, the Henry's law constant is derived from solubility expressed as the mole fraction (*x*). Due to their low or negligible vapor pressure, ILs are considered to represent a pure gas solute in the gas phase.^47,64^ The fugacity coefficient is assumed to be unity, and the Henry's law constant (*k*_H_) is found using the following equation:^65–68^1
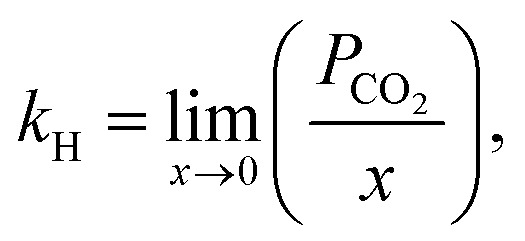
where *P* is the partial pressure of the gas and *k*_H_ (*T*) will have units of pressure and is inversely proportional to the mole fraction of the gas in the liquid (*x*). For gases that behave almost ideally, solubility is proportional to pressure. Calculating the linear slope of the data yields the Henry's law constant.

**Fig. 6:**
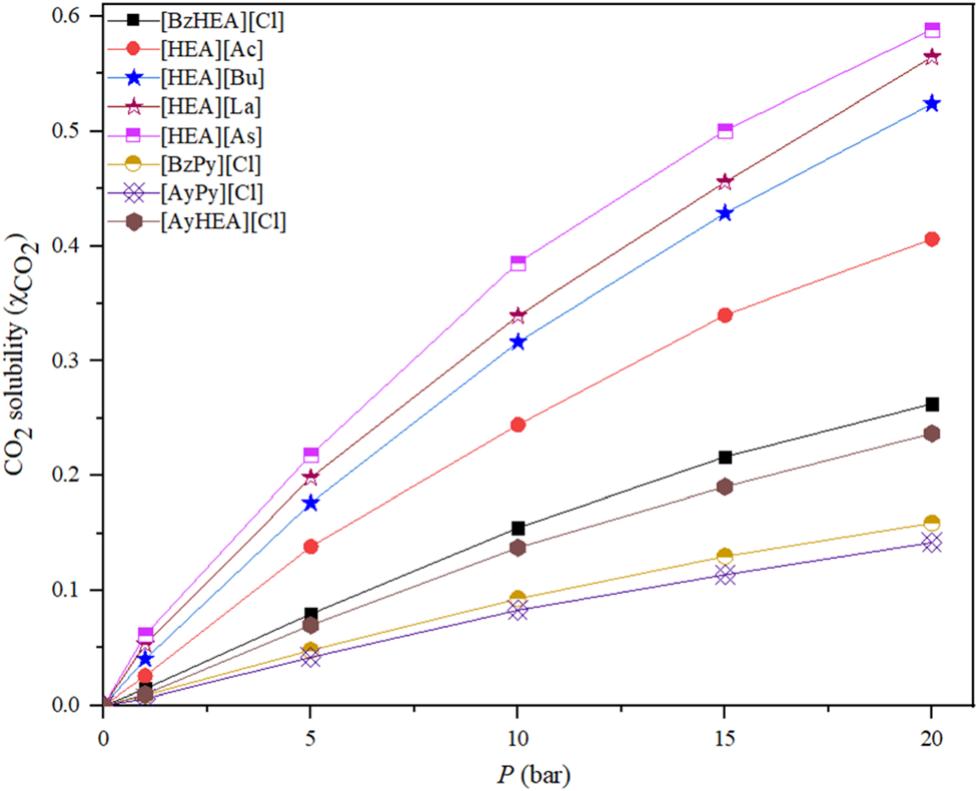
Pressure *versus* mole fraction of CO_2_ for the prepared ILs at 298.15 K.

The CO_2_ gas exhibits a nonlinear trend as the CO_2_ pressure increases for all the studied ILs, as shown in Fig. 6. (The curves begin to flatten out, indicating that the IL approaches its maximum, pressure-independent capacity for CO_2_.^69^) So, the Henry's law constant can be found by fitting a second-order polynomial to the data and calculating the limiting slope as the solubility approaches zero.^65,70^

Accordingly, eqn (2) was used to model the experimental values,^71^ with a correlation coefficient (*R*^*2*^) greater than 0.996.

2 *k*_H_ = *ax*^2^ + *bx* + *c*

Moreover, Henry's law constants may be utilized for classifying the absorption as either physical or chemical in nature. A Henry's law constant value below 3 MPa at 298 K often indicates the chemical absorption of CO_2_ into ILs.^72^

The Henry's law constants for all investigated ILs, together with the correlation coefficients of the polynomial equation (eqn (2)), are shown in Table 3. The Henry's law constant indicates gas solubility in a solvent; a decline in its value indicates an increase in gas solubility in the solvent.

Henry's law constant and correlation coefficients for the studied ILs

**Table:** 

Property	[HEA][As]	[HEA][La]	[HEA][Bu]	[HEA][Ac]
*k*_H_ (298.15 K)	13.01	20.58	25.02	27.08
*R* ^2^	0.999	0.999	0.998	0.999

**Table:** 

	[BzHEA][Cl]	[AyHEA][Cl]	[BzPy][Cl]	[AyPy][Cl]
*k*_H_ (298.15 K)	42.56	56.96	70.93	94.90
*R* ^2^	0.996	0.999	0.996	0.999

**Fig. 7:**
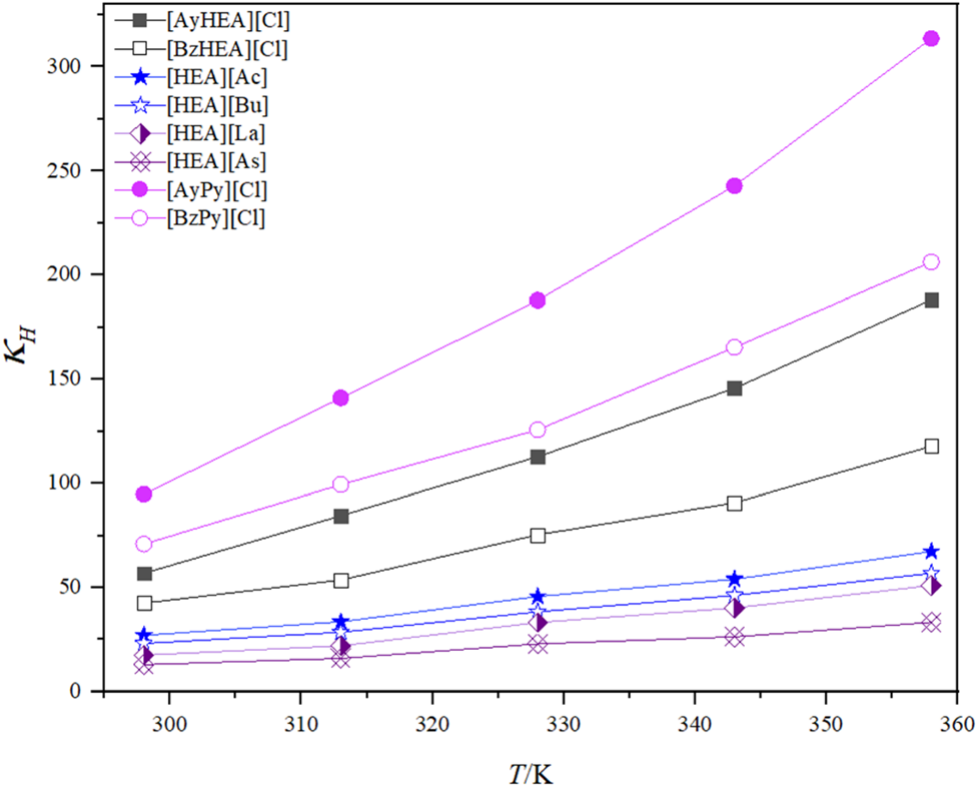
Effect of temperature on CO_2_ solubility in terms of Henry's law constant for the prepared ILs.

According to the study, the ILs with the [As] anion have the lowest Henry's law constant value among the studied [HEA] cation-based ILs, whereas the ILs with the [BzHEA] cation have the lowest value among the studied [Cl]-based ILs. In addition, the effect of temperature on these ILs' CO_2_ dissolution capacity was investigated using the Henry's law constant. The Henry's law constants were calculated for the synthesized ILs at pressures of 1, 5, 10, 15, and 20 bar and temperatures of 298.15, 313.15, 328.15, 343.15, and 358.15 K using eqn (1), as shown in Fig. 7.

The solubility of CO_2_ in ILs decreases as the temperature increases, as illustrated in Fig. 7, which is consistent with the reported results. Increasing the temperature causes an increase in kinetic energy, which in turn causes ions to move more quickly. Because of this, the spacing between ions decreases, which may lead to an increase in the uneven distribution of ions.

#### 3.2.4 Enthalpy, entropy and Gibbs free energy

The solubility properties were explained by the thermodynamic excess functions: standard enthalpy (Δ*H*^0^), entropy (Δ*S*^0^), and Gibbs free energy (Δ*G*^0^). Significantly negative values for excess Gibbs energy frequently suggest the formation of chemical complexes, while the heat of mixing (or excess enthalpy) is more negative than the TSE.^23^ The standard entropy (Δ*S*^0^) of the ionic liquid–gas combination indicates the level of order in the system, whereas the standard enthalpy (Δ*H*^0^) of solvation denotes the intensity of contact between the liquid and the gas. The Gibbs free energy (Δ*G*^0^) indicates the spontaneity of gas dissolution in a liquid and the phase stability of the system. Moreover, negative Δ*G*^0^ values frequently indicate the production of one or more chemical complexes.

At infinite dilution, the temperature dependence of Henry's law constants serves as a foundation for evaluating their role in gas solvation inside a solvent. The standard enthalpy, entropy, and Gibbs free energy of gas solubility may be derived from these constants.^45,49,73^ Furthermore, eqn (3) employs the standard pressure, *P*^0^ = 1.01325 bar, and Henry's law constant to calculate the standard Gibbs free energy of gas dissolution.^6^3
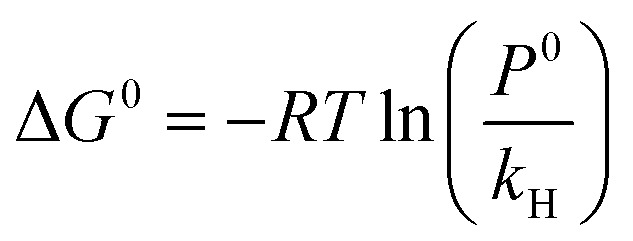


The standard entropy of the ionic liquid–gas mixture represents the degree of order in the system, whereas the standard enthalpy of solvation represents the intensity of the contact between the liquid and the gas.^73,74^ Gibbs free energy influences phase stability as well as the spontaneous dissolution of gases in liquids. Additionally, negative Δ*G*^0^ readings frequently indicate the production of one or more chemical complexes.^75^

In certain cases, the standard heat of solution of a gas (Δ*H*^0^) may be treated as a constant (for a small temperature change) and can be related to the Henry's law constant at infinite dilution using the linear equation:4
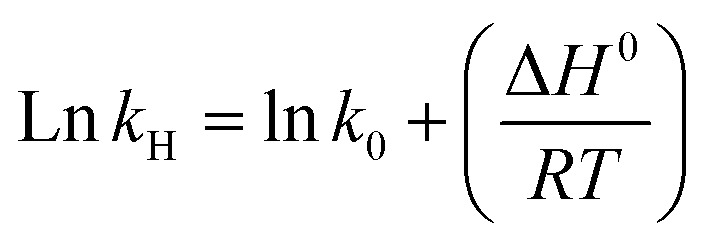


However, there are other cases (for relatively wide temperature ranges) in which Δ*H*^0^ is temperature-dependent and, therefore, is not a constant. For these cases, Δ*H*^0^ may be obtained from eqn (5).^67^5
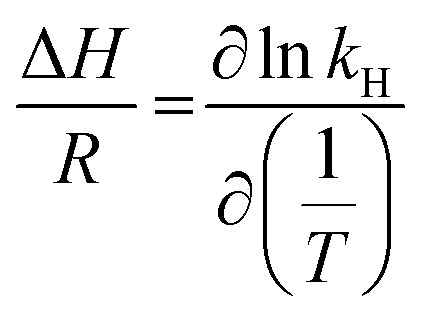


The change in molar enthalpy upon gas solubility was obtained by plotting the natural logarithm of the calculated Henry constant (ln *k*_H_) *versus* the reciprocal of the absolute temperature 
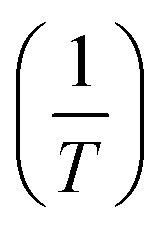
, and the curve was fitted with the following equation:^67^6
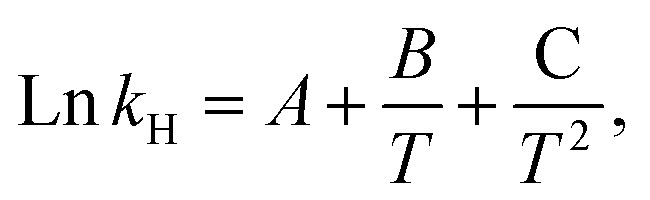
where *A, B* and *C* are correlation coefficients. A combination of eqn (5) and (6) was used to estimate the standard enthalpy using the following equation:^67^7
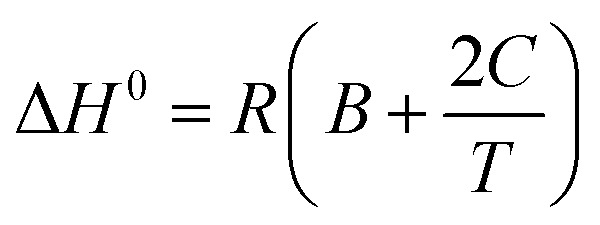


The standard entropy was calculated using the following equation:^67^8
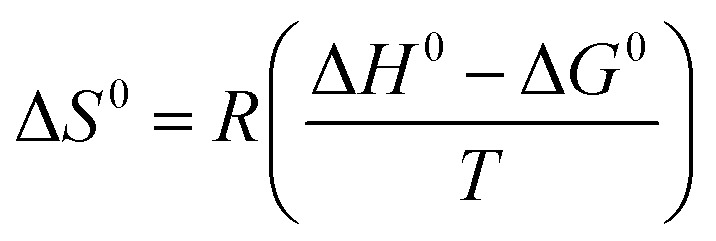


The standard enthalpy (Δ*H*^0^), entropy (Δ*S*^0^) and Gibbs free energy (Δ*G*^0^) of the dissolution of CO_2_ in the prepared ILs at different temperatures are given in Table 4. As indicated above, based on the relationship given in eqn (1) and the data presented in Fig. 7, *k*_H_ increases with temperature, which corresponds to a decrease in the mole fraction of CO_2_ (*X*_CO_2__) in the ILs. According to this behavior, the solubility of CO_2_ diminishes at elevated temperatures. The positive values of Δ*G*^0^ show that CO_2_ dissolution in the examined ILs is non-spontaneous in the temperature range of 298.15–358.15 K.^67^ Concurrently, the negative values of Δ*H*^0^ verify that CO_2_ continues to dissolve in these systems throughout this range.^67^ The magnitude of Δ*H*^0^ for [HEA][Bu], [AyHEA][Cl], [BzHEA][Cl], [AyPy][Cl], and [BzPy][Cl] changes from values corresponding to moderately strong acid–base interactions at 298.15 K to values representative of weak acid–base interactions at 358.15 K, whereas the changes for [HEA][As], [HEA][Ac], and [HEA][La] are quite minor in comparison. Both the enthalpy of condensation of the pure gas and the partial enthalpy of mixing between the condensed gas and the liquid phase can be considered components of the partial molar enthalpy change, respectively.^67^

**Table 4:** Thermodynamic properties for the dissolution of CO_2_ in the synthesized ILs

Thermodynamic property	IL	Temperature (K)				
		298.15	313.15	328.15	343.15	358.15
Δ*G*^0^ (kJ mol^−1^)	[AyHEA][Cl]	9.99	11.52	12.86	14.18	15.56
	[BzHEA][Cl]	9.27	10.33	11.76	12.83	14.16
	[HEA][Ac]	8.14	9.12	10.40	11.35	12.50
	[HEA][Bu]	7.97	8.70	9.92	10.90	12.00
	[HEA][La]	7.46	8.45	9.76	10.71	11.85
	[HEA][As]	6.33	7.18	8.52	9.29	10.40
	[AyPy][Cl]	11.25	12.85	14.25	15.63	17.08
	[BzPy][Cl]	10.53	11.66	13.15	14.29	15.68
Δ*H*^0^ (kJ mol^−1^)	[AyHEA][Cl]	−19.68	−18.39	−17.21	−16.14	−15.16
	[BzHEA][Cl]	−13.97	−14.66	−15.29	−15.86	−16.38
	[HEA][Ac]	−13.28	−13.47	−13.65	−13.81	−13.96
	[HEA][Bu]	−8.99	−10.98	−12.79	−14.43	−15.94
	[HEA][La]	−14.67	−14.56	−14.45	−14.36	−14.27
	[HEA][As]	−14.25	−14.17	−14.09	−14.03	−13.96
	[AyPy][Cl]	−19.70	−18.40	−17.22	−16.14	−15.15
	[BzPy][Cl]	−13.98	−14.66	−15.29	−15.86	−16.38
Δ*S*^0^ (J mol^−1^ K^−1^)	[AyHEA][Cl]	−99.51	−95.51	−91.63	−88.35	−85.76
	[BzHEA][Cl]	−77.95	−79.83	−82.43	−83.60	−85.28
	[HEA][Ac]	−71.85	−72.13	−73.28	−73.31	−73.88
	[HEA][Bu]	−56.88	−62.84	−69.19	−73.83	−78.01
	[HEA][La]	−74.24	−73.48	−73.78	−73.06	−72.93
	[HEA][As]	−69.00	−68.17	−68.91	−67.95	−68.03
	[AyPy][Cl]	−103.81	−99.79	−95.89	−92.59	−89.99
	[BzPy][Cl]	−82.20	−84.08	−86.67	−87.84	−89.52

The enthalpy of a liquid is often lower than that of a gas, resulting in a negative enthalpy change during gas condensation. The magnitude and sign of the enthalpy of mixing dictate the sign and size of the total partial enthalpy change related to solvation, respectively. In gas–liquid systems with elevated solubility, the enthalpy of condensation prevails, leading to a net negative partial molar enthalpy change.^10,33^ Furthermore, lower enthalpy values signify enhanced interactions between the IL and CO_2_,^35^ indicating that [HEA][Bu] demonstrates stronger interactions with CO_2_ compared to other prepared ILs, and the ILs incorporating the benzyl group show stronger interactions than those incorporating the allyl group, but the temperature's influence on this interaction is less pronounced for [HEA][Ac], [HEA][La], and [HEA][As]. The negative enthalpies of solvation signify that the solvation process is exothermic.

The rise in Δ*G*^0^ as *T* rises shows that the process of CO_2_ dissolution takes more energy as the temperature rises.^67^ Among the studied ILs, CO_2_ solubility in the ILs incorporating allyl and benzyl groups shows an unfavorable change in entropy (Δ*S*^0^) between 298.15 and 358.15 K^67^ compared to those incorporating [Ac], [By], [La], and [As] anions, which display a relatively small change compared to the other studied ILs.

The negative entropy of solvation can be ascribed to the structuring effect resulting from particular interactions between the solute and the charged sites of the ionic liquid. The apparent increase in solubility is determined by the equilibrium between CO_2_–ionic liquid interactions (represented by the enthalpic contribution) and the organization of the solvent environment surrounding the solute (represented by the entropic contribution). Molecular simulation studies have also revealed that negative solvation entropy values are caused by interactions between the solute and the charged centers of the ionic liquid.^76^

The studied ILs have negative standard enthalpies (Δ*H*^0^ = −9 to −20 kJ mol^−1^) and entropies (Δ*S*^0^ = −57 to −104 J mol^−1^ K^−1^) but positive Gibbs free energies (Δ*G*^0^ = 6–11 kJ mol^−1^), indicating exothermic physisorption caused by entropy loss during CO_2_ dissolution. The magnitude of the absolute value of Δ*H*^0^ is significantly smaller than that reported for chemisorbing task-specific ILs (TSILs, typically −40 to −80 kJ mol^−1^), confirming the non-reactive, reversible nature of absorption and the low energy penalty for regeneration—a key advantage for pressure-swing processes.^77,78^

## 4. Conclusion

This study revealed that the structures of both cations and anions significantly influence the CO_2_ capture properties of the produced ILs. The experimental findings validated that CO_2_ solubility rises with pressure across all ILs, but elevated temperatures diminish solubility and improve Henry's law constants, signifying a decreased gas affinity at higher temperatures. Among the examined anions, the [As]-based ILs exhibit the maximum CO_2_ absorption, while among the [Cl]-based ILs, the [Bz]-based ILs outperform their [Ay] counterparts. These patterns underscore the significance of the molecular architecture, free volume, and particular ion–gas interactions in regulating absorption efficacy.

The thermodynamic study indicated that CO_2_ solvation is exothermic, as evidenced by negative Δ*H*^0^ values, while the positive Δ*G*^0^ values suggest that dissolution is non-spontaneous over the examined temperature range. The observed changes in entropy indicate that solvation is affected by variations in IL ordering and the arrangement of the solvent surrounding the gas molecule. The findings affirm that the structural modification of ILs is a highly effective method for adjusting CO_2_ solubility and thermodynamic properties. These findings offer valuable insights for the development of next-generation ILs with enhanced efficacy in carbon capture applications.

## Conflicts of interest

There are no conflicts to declare.

## Supplementary Material

RA-016-D6RA03083A-s001

## Data Availability

The data that support our findings are available in the text of this article and the supplementary information (SI). Supplementary information is available. See DOI: https://doi.org/10.1039/d6ra03083a.
