# Supplementary material for: The role of cation and anion structural modifications for the enhanced CO2 solubility of hydroxyl ammonium- and pyridinium-based ionic liquids
Source: RSC Adv. 2026 May 26;16(31):28265–79. doi: 10.1039/d6ra03083a (PMC13213566; doi:10.1039/d6ra03083a)
Supplement: RA-016-D6RA03083A-s001 [file RA-016-D6RA03083A-s001.pdf]

Supplementary Information (SI) for RSC Advances.
This journal is © The Royal Society of Chemistry 2026

Electronic Supplementary Information

The Role of Cation and Anion Structural Modifications for Enhanced CO_2 Solubility of hydroxyl ammonium and pyridinium-based Ionic Liquid

Ahmed Mohamed Abdelmagid[1], Abobakr Khidir Ziyada [1,2], Abdalla Ahmed Elbashir[3*], Zakaria A. Salih[4]*

[1]Department Chemical Engineering and Chemical Technology, Faculty of Engineering and Technology, University of Gezira, Wad Medani, 21113, Sudan

[2] General Studies Department, Jubail Industrial College, Jubail Industrial City 31961, Saudi Arabia

[3]Department of Chemistry, College of Science, King Faisal University, P.O.Box 400, Al-Ahsa, 31982, Saudi Arabia.

[4]Research and Training Station, King Faisal University, P.O.Box 400, Al-Ahsa, 31982, Saudi Arabia.

*All correspondence should be addressed to Ahmed Mohamed Abdelmagid and Abdalla Ahmed Elbashir

* Authors whom all correspondence should be addressed.

Email: bahooti@gmail.com

E mail: aaeahmed@kfu.edu.sa

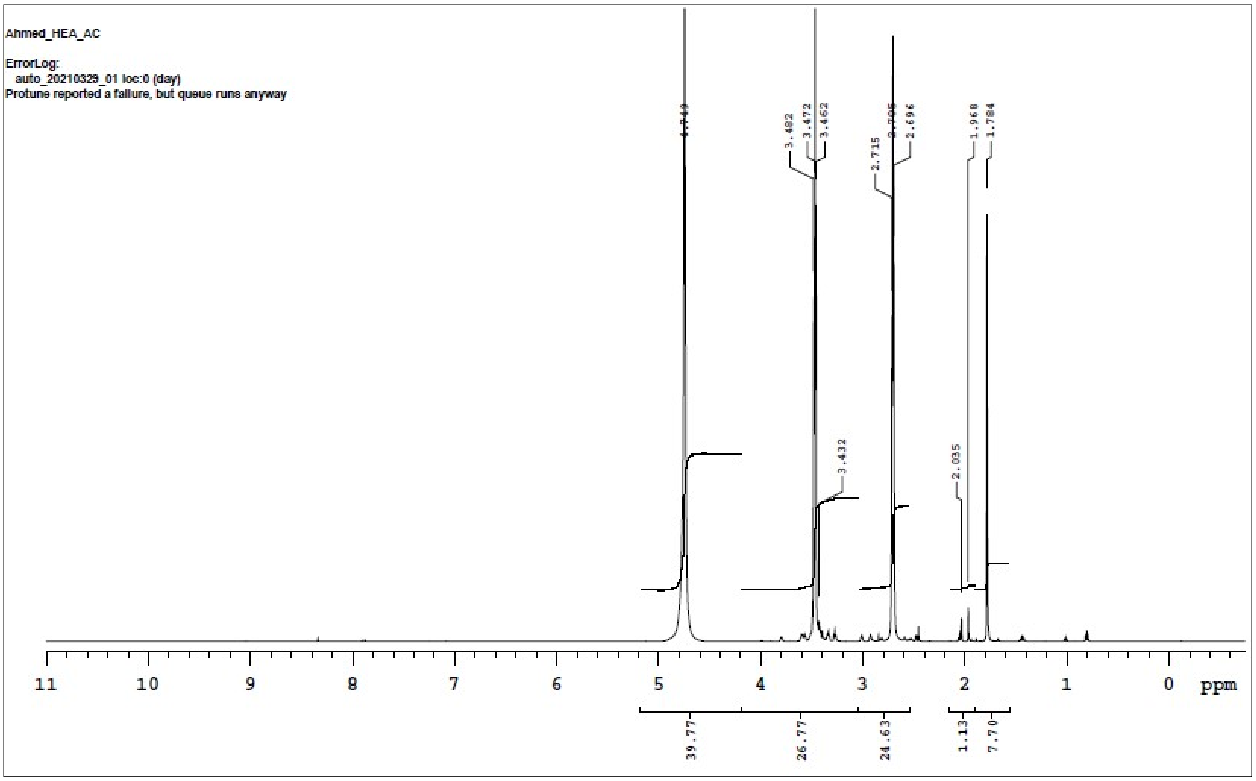

Figure S.1. ^{1}H NMR spectra for [HEA][Ac].

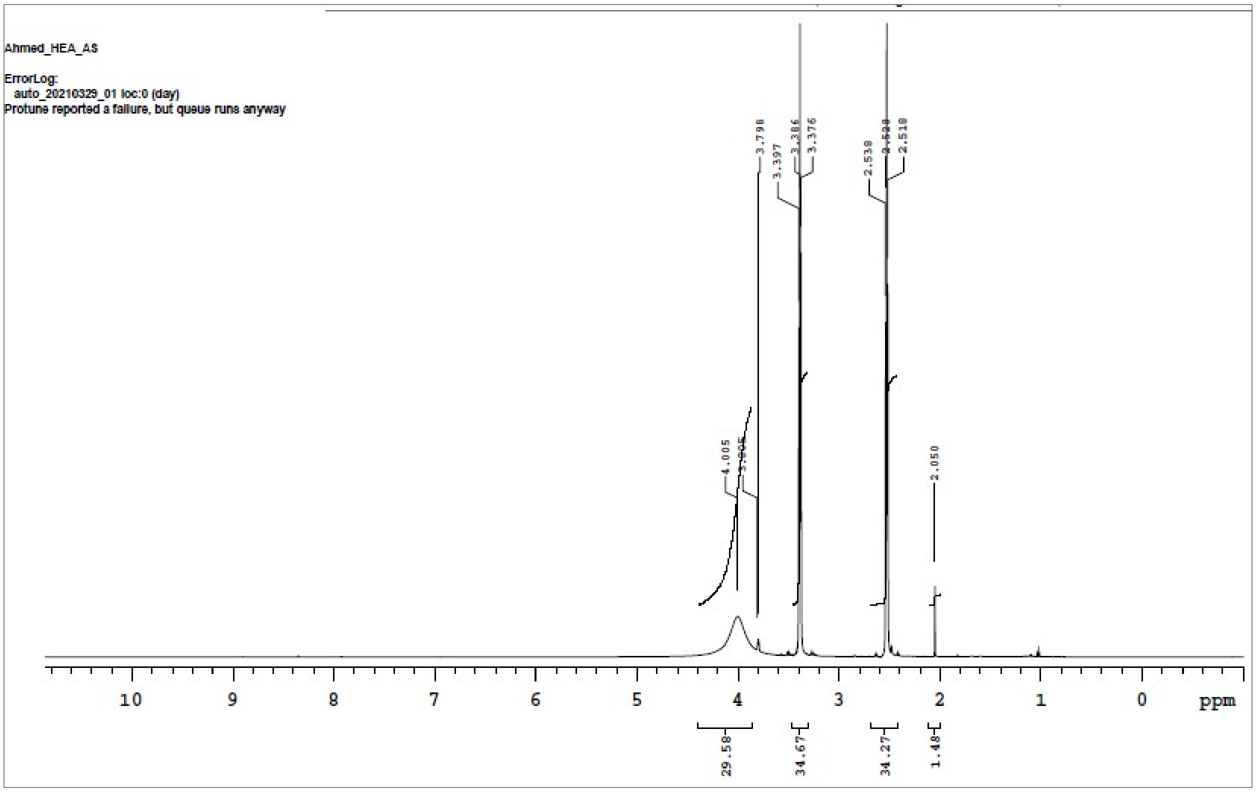

Figure S.2. ^{1}H NMR spectra [HEA][As]

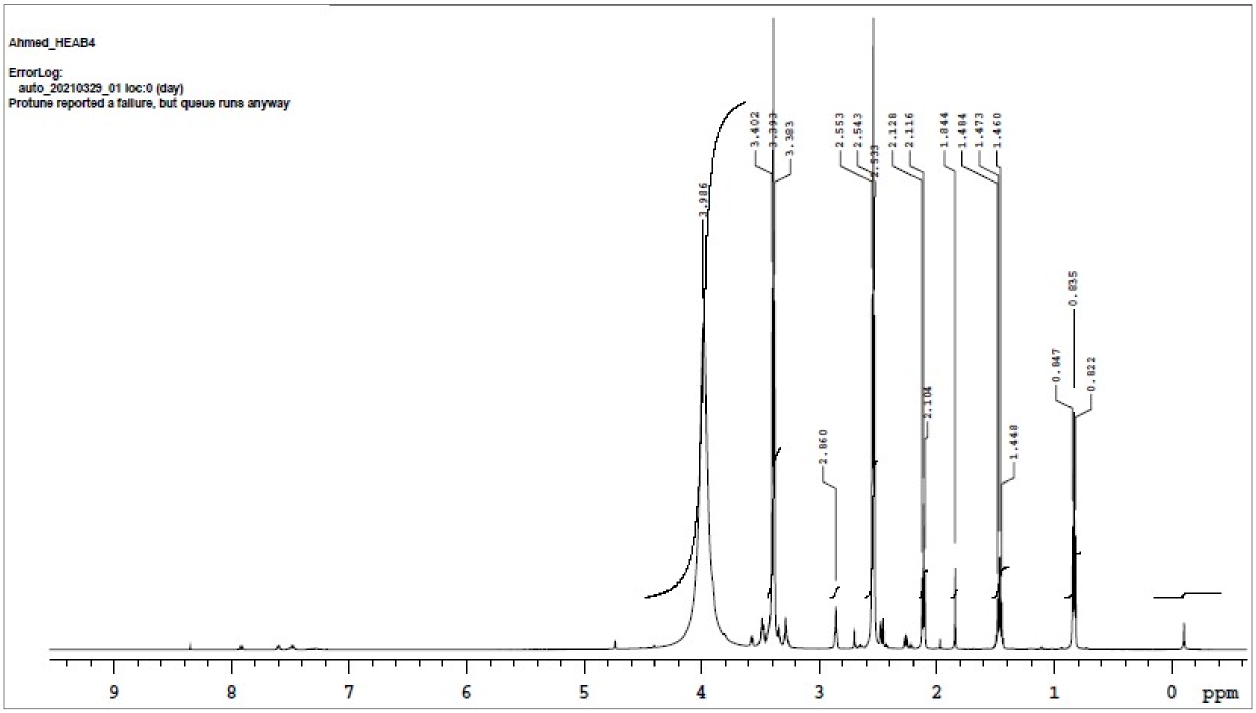

Figure S.3. ^{1}H NMR spectra for [HEA][Bu].

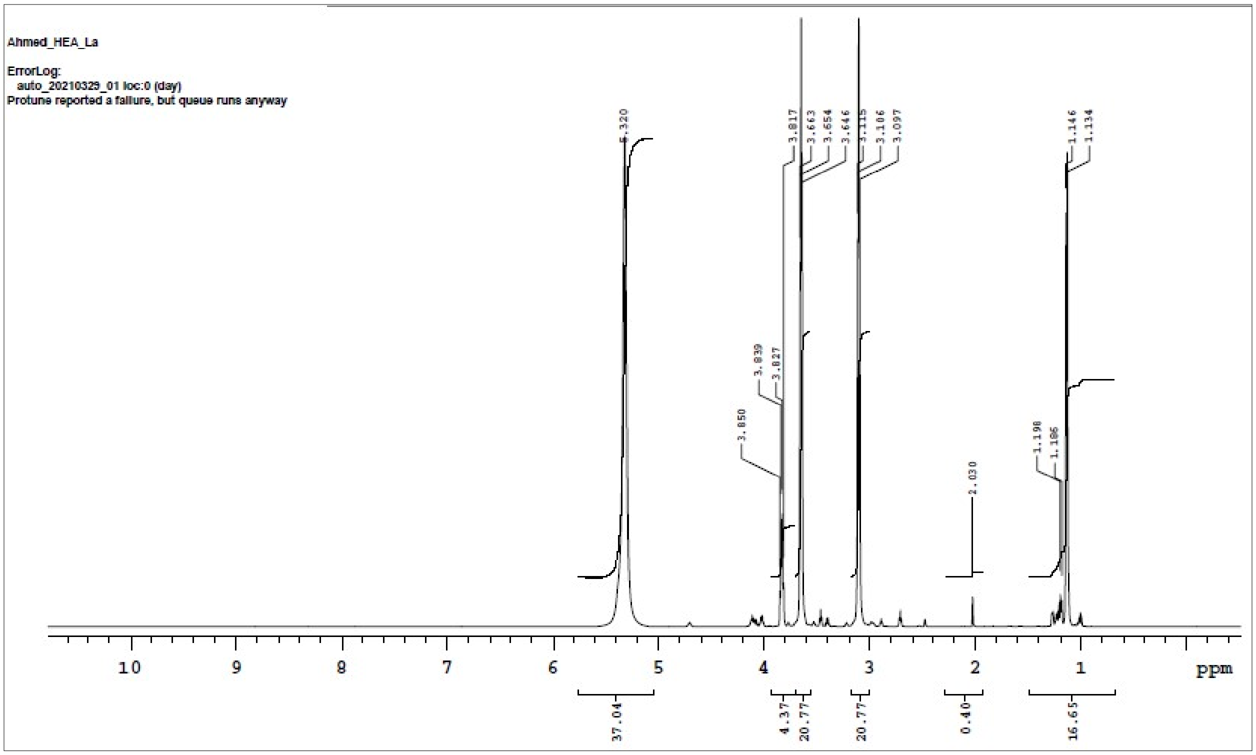

Figure S.4. ^{1}H NMR spectra for [HEA][La].

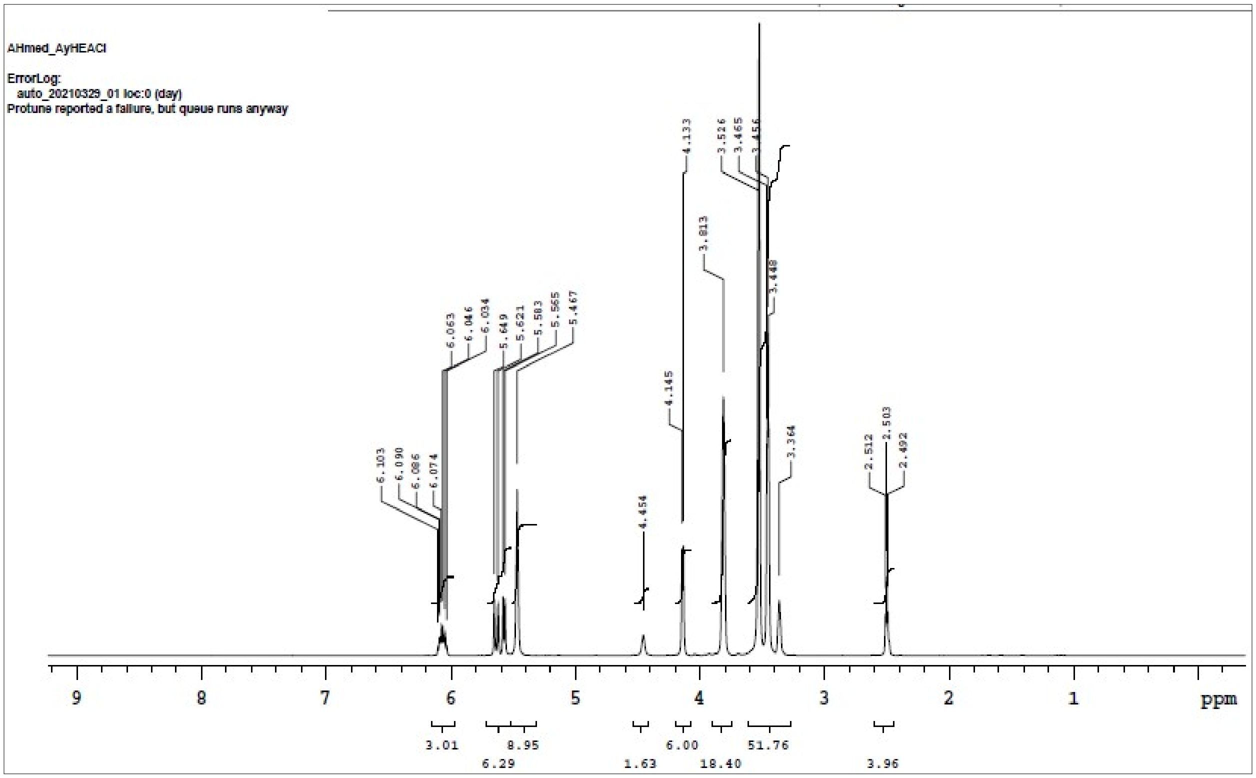

Figure S.5. ^{1}H NMR spectra for [AyHEA][Cl].

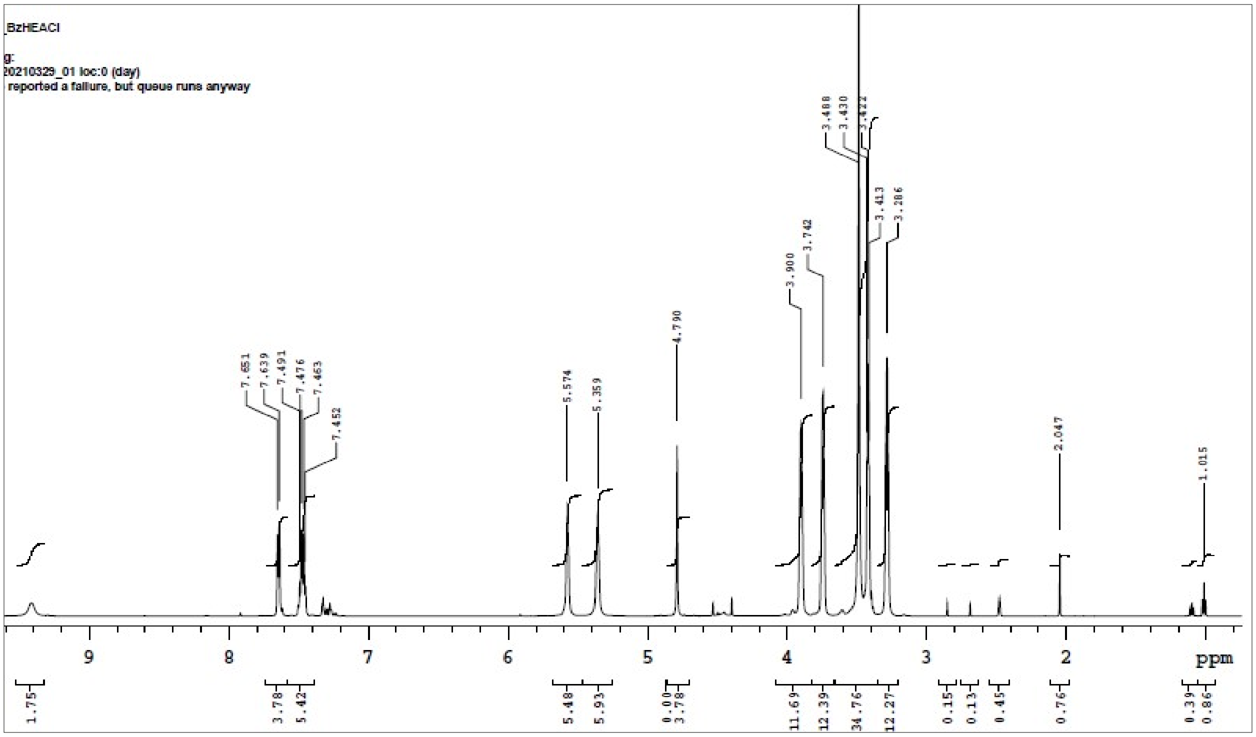

Figure S.6. ^{1}H NMR spectra for [BzHEA][Cl].

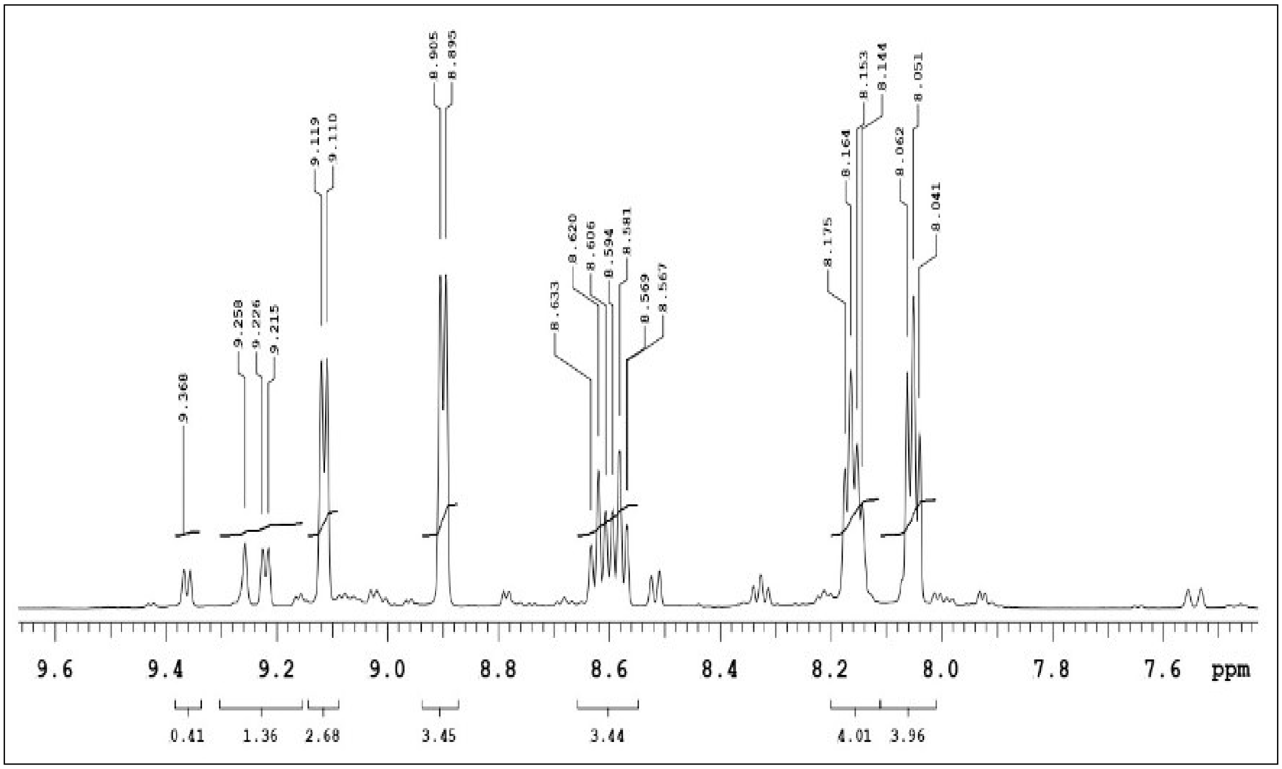

Fig S. 7. ^{1}H NMR spectra for [AyPy][Cl]

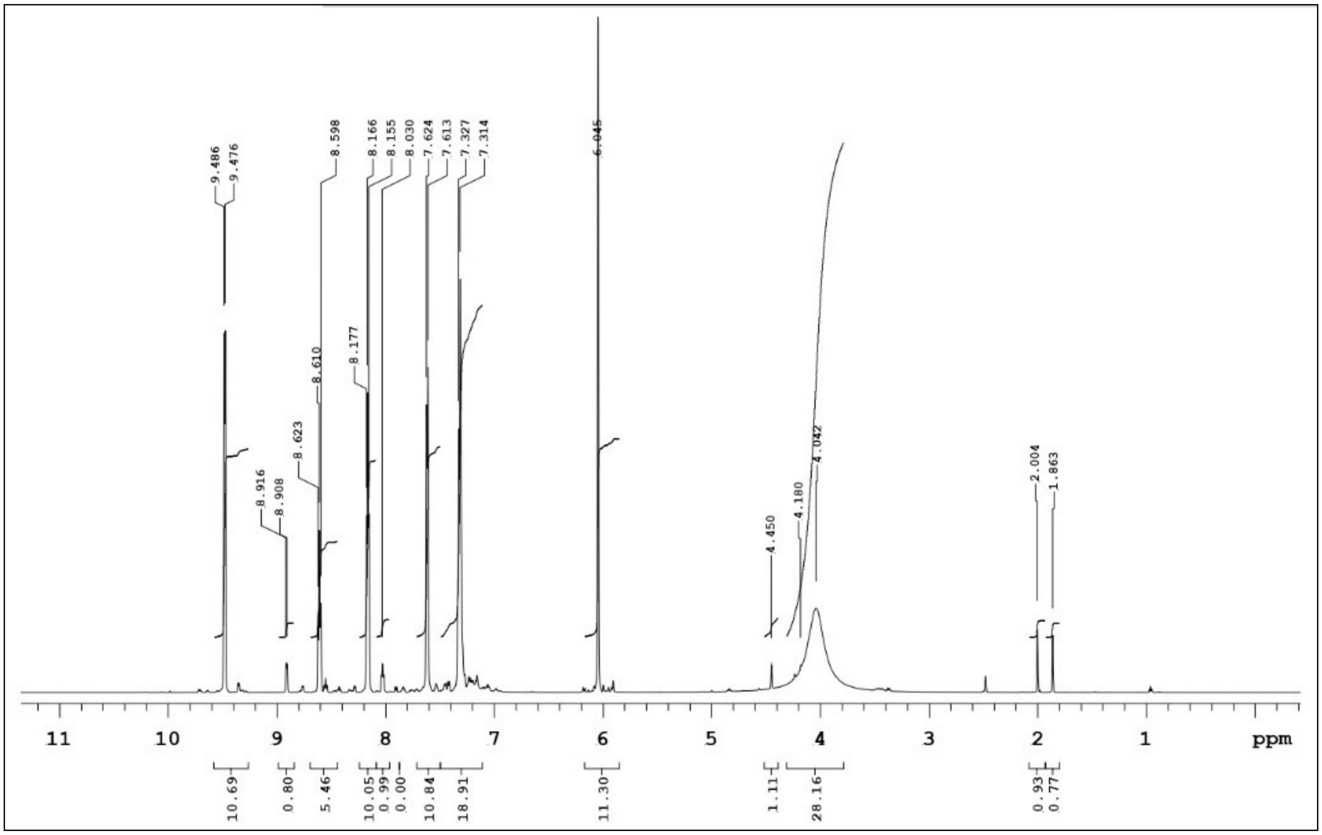

Fig S. 8. ^{1}H NMR spectra for [BzPy][Cl]

Table S. 1. Experimental solubility data for CO_2 in [HEA][As] at different pressures

Time (min)	20 bar χ	Time (min)	15 bar χ	Time (min)	10 bar χ	Time (min)	5 bar χ	Time (min)	1 bar χ
0	0	0	0	0	0	0	0	0	0
3.6	0.200	4.41	0.117	3.05	0.080	2.6	0.050	5.76	0.021
9	0.293	6.3	0.149	4.27	0.106	5.2	0.064	12.8	0.031
12.6	0.332	10.71	0.212	7.93	0.145	8.45	0.091	16.64	0.035
18	0.398	17.01	0.274	14.03	0.205	13.65	0.113	21.76	0.042
28.2	0.462	20.79	0.303	20.13	0.246	21.45	0.131	30.08	0.049
41.4	0.513	23.94	0.330	25.62	0.276	29.25	0.151	36.48	0.054
48	0.533	35.28	0.389	36.6	0.316	38.35	0.164	39.04	0.056
58.2	0.551	46.62	0.423	41.48	0.337	57.2	0.184	44.16	0.058
65.4	0.556	52.29	0.432	46.97	0.346	77.35	0.192	50.56	0.060
78	0.563	66.78	0.456	54.9	0.359	92.3	0.196	62.72	0.061
91.2	0.568	74.34	0.461	66.49	0.366	117	0.204	69.76	0.061
100.8	0.574	83.16	0.470	82.35	0.374	120.9	0.205	80.64	0.061
117.6	0.578	90.72	0.476	98.82	0.383	128.7	0.206	88.32	0.061
126.6	0.580	98.91	0.483	106.75	0.385	133.9	0.208	96	0.062
129	0.581	115.29	0.492	120.78	0.385	138.45	0.209	112	0.062
137.4	0.583	123.48	0.497	125.66	0.385	144.95	0.213	119.68	0.062
145.8	0.588	131.04	0.498	142.13	0.385	153.4	0.214	135.04	0.062
154.2	0.588	147.42	0.500	147.62	0.385	157.3	0.215	143.36	0.062
168.6	0.588	171.99	0.501	--	--	168.35	0.218	161.28	0.062

Table S. 2. Experimental solubility data for CO_2 in [HEA][La] at different pressures

Time (min)	20 bar χ	Time (min)	15 bar χ	Time (min)	10 bar χ	Time (min)	5 bar χ	Time (min)	1 bar χ
0	0	0	0	0	0	0	0	0	0
1.2	0.117	2.52	0.068	5	0.083	3.25	0.040	1.28	0.011
2.4	0.155	4.41	0.097	8	0.117	5.85	0.053	2.56	0.015
6.6	0.212	6.93	0.129	11	0.160	9.1	0.072	7.04	0.020
14.4	0.300	10.08	0.176	13	0.173	14.95	0.102	14.72	0.028
21	0.361	18.27	0.246	16	0.198	27.3	0.131	21.76	0.034
27	0.404	23.31	0.271	19	0.222	31.85	0.138	28.16	0.038
37.8	0.463	32.76	0.317	21.96	0.238	39	0.149	39.68	0.044
43.8	0.493	36.54	0.331	24.4	0.250	46.15	0.160	45.44	0.046
49.2	0.507	39.69	0.338	28.67	0.269	52	0.170	51.2	0.048
57.6	0.526	43.47	0.346	35.38	0.286	59.15	0.176	60.16	0.049
69	0.541	61.11	0.385	43.92	0.305	62.4	0.180	71.68	0.050
86.4	0.553	69.3	0.400	53.07	0.315	77.35	0.185	89.6	0.052
94.8	0.559	83.16	0.416	61.61	0.324	88.4	0.190	98.56	0.052
103.2	0.562	106.47	0.432	70.76	0.330	99.45	0.191	107.52	0.053
111.6	0.564	120.96	0.444	84.18	0.336	110.5	0.192	116.48	0.053
126	0.564	129.15	0.450	99.43	0.338	114.4	0.192	131.2	0.053
131.4	0.564	143.01	0.455	114.68	0.339	125.45	0.192	136.96	0.053
148.2	0.564	153.09	0.456	132.37	0.339	136.5	0.192	154.24	0.053
165	0.564	161	0.456	--	--	161.2	0.198	157.44	0.053

Table S. 3. Experimental solubility data for CO_2 in [HEA][Bu] at different pressures

Time (min)	20 bar χ	Time (min)	15 bar χ	Time (min)	10 bar χ	Time (min)	5 bar χ	Time (min)	1 bar χ
0	0	0	0	0	0	0	0	0	0
3.6	0.132	3.15	0.088	3.66	0.074	4.55	0.036	1.92	0.010
6	0.186	5.67	0.117	6.1	0.095	7.8	0.048	3.84	0.014
11.4	0.255	9.45	0.161	9.15	0.135	12.35	0.066	6.4	0.019
13.8	0.275	18.27	0.228	14.03	0.174	18.2	0.094	12.16	0.023
17.4	0.315	27.09	0.274	17.69	0.193	24.7	0.113	16	0.026
22.2	0.353	34.65	0.307	21.35	0.210	31.85	0.126	22.4	0.030
25.8	0.380	38.43	0.319	25.01	0.230	40.95	0.145	32	0.034
34.2	0.428	44.1	0.329	36.6	0.261	47.45	0.154	40.32	0.036
39.6	0.455	49.77	0.345	42.09	0.268	53.3	0.159	56.32	0.038
47.4	0.486	57.96	0.363	47.58	0.274	62.4	0.164	64.64	0.039
58.2	0.516	69.93	0.377	67.1	0.290	74.75	0.168	76.16	0.040
63.6	0.525	86.94	0.401	75.64	0.295	93.6	0.171	90.24	0.040
75.6	0.534	95.76	0.416	84.18	0.300	102.7	0.174	104.32	0.040
89.4	0.538	103.95	0.421	86.62	0.303	111.8	0.176	120.32	0.040
103.2	0.540	112.77	0.428	95.16	0.307	120.9	0.176	126.08	0.040
118.8	0.540	126.63	0.428	111.63	0.314	136.5	0.176	133.76	0.040
150.6	0.544	132.3	0.429	120.17	0.315	142.35	0.176	138.88	0.040
155.4	0.544	149.31	0.429	136.64	0.317	150.15	0.176	144	0.040
160.8	0.545	154	0.429	--	--	153.4	0.176	146.56	0.040

Table S.4. Experimental solubility data for CO_2 in [HEA][Ac] at different pressures

Time (min)	20 bar χ	Time (min)	15 bar χ	Time (min)	10 bar χ	Time (min)	5 bar χ	Time (min)	1 bar χ
0	0	0	0	0	0	0	0	0	0
4.2	0.109	4.41	0.081	4.27	0.025	3.25	0.020	1.92	0.003
7.2	0.164	12	0.175	10.37	0.085	10.4	0.056	5.12	0.008
9.6	0.213	19.53	0.235	14.64	0.118	16.9	0.081	10.24	0.012
18	0.297	28.98	0.264	18.3	0.143	24.05	0.099	27.52	0.018
24.6	0.328	40.95	0.282	28.06	0.180	31.85	0.112	39.04	0.021
33	0.357	51.03	0.291	37.21	0.207	50.7	0.126	53.76	0.023
42.6	0.379	56.07	0.296	43.92	0.216	55.25	0.129	65.28	0.024
51.6	0.392	64.89	0.306	55.51	0.226	68.25	0.132	71.04	0.025
61.8	0.395	68.67	0.310	67.1	0.233	70.2	0.134	76.16	0.025
72.6	0.398	74.97	0.317	71.98	0.235	78	0.135	80.64	0.025
81	0.402	80.64	0.321	78.69	0.238	81.25	0.136	87.68	0.025
92.4	0.403	86.94	0.327	90.28	0.240	87.1	0.136	92.16	0.025
102	0.405	92.61	0.328	98.21	0.241	94.25	0.137	98.56	0.025
105.6	0.405	98.91	0.330	101.26	0.242	99.45	0.138	103.68	0.025
112.8	0.406	113.4	0.331	112.85	0.244	117.65	0.138	110.72	0.026
115.8	0.406	117.18	0.333	118.34	0.244	122.85	0.138	114.56	0.026
123.6	0.406	122.85	0.337	122	0.244	126.75	0.138	118.4	0.026
130.8	0.406	131.67	0.338	129.32	0.244	130.65	0.138	122.24	0.026
135	0.406	135	0.340	--	--	135.2	0.138	128	0.026

Table S. 5. Experimental solubility data for CO_2 in [BzHEA][Cl] at different pressures

Time (min)	20 bar χ	Time (min)	15 bar χ	Time (min)	10 bar χ	Time (min)	5 bar χ	Time (min)	1 bar χ
0	0	0	0	0	0	0	0	0	0
1.8	0.041	6	0.085	2.4	0.019	7.8	0.031	5.4	0.004
5.4	0.110	13	0.136	10.2	0.073	12.6	0.042	10.2	0.007
12.6	0.172	18	0.156	15.6	0.093	19.2	0.053	14.4	0.009
15	0.182	23	0.177	24	0.113	27	0.062	19.8	0.010
19.8	0.200	32	0.189	32.4	0.125	32.4	0.066	33	0.011
28.2	0.213	40.2	0.198	43.2	0.132	40.8	0.071	37.2	0.011
45.6	0.228	46.2	0.202	54.6	0.137	51.6	0.072	40.8	0.012
52.8	0.232	54	0.205	67.8	0.138	57.6	0.073	45	0.012
61.2	0.236	58.8	0.208	74.4	0.142	64.2	0.074	51.6	0.012
66.6	0.237	69.6	0.209	85.2	0.144	70.8	0.075	54	0.013
79.2	0.242	76.8	0.211	90	0.146	81	0.075	61.8	0.013
87	0.244	85.8	0.212	103.2	0.146	85.2	0.076	64.8	0.013
97.2	0.246	96.6	0.213	107.4	0.147	98.4	0.076	71.4	0.013
109.2	0.248	99.6	0.213	118.8	0.152	102.6	0.077	75	0.013
118.2	0.250	113.4	0.216	125.4	0.154	112.8	0.078	86.4	0.014
128.4	0.252	136.8	0.216	131.4	0.155	119.4	0.078	96.6	0.014
140.4	0.252	137.4	0.216	136.2	0.155	123.6	0.079	113.4	0.014
147.6	0.252	144	0.216	139.2	0.155	140.4	0.081	125.4	0.015
153	0.252	148.8	0.217			143.4	0.080	141.6	0.015

Table S.6. Experimental solubility data for CO_2 in [AyHEA][Cl] at different pressures

Time (min)	20 bar χ	Time (min)	15 bar χ	Time (min)	10 bar χ	Time (min)	5 bar χ	Time (min)	1 bar χ
0	0	0	0	0	0	0	0	0	0
1.8	0.027	4.2	0.054	2.4	0.013	3	0.004	9.6	0.004
3	0.050	10.2	0.095	9.6	0.054	6.6	0.009	14.4	0.005
6	0.086	12.6	0.107	12.6	0.069	10.8	0.015	17.4	0.006
10.2	0.119	16.2	0.123	17.4	0.083	14.4	0.020	27	0.007
12.6	0.139	21	0.138	22.8	0.095	18.6	0.025	40.2	0.007
21	0.170	25.2	0.149	30.6	0.107	25.8	0.034	44.4	0.007
31.2	0.192	30.6	0.161	37.8	0.113	31.2	0.042	48	0.007
35.4	0.200	38.4	0.169	48	0.118	34.2	0.045	52.2	0.008
47.4	0.206	43.8	0.173	58.8	0.122	39.6	0.051	58.8	0.008
60.6	0.213	48	0.176	63.6	0.122	45	0.054	61.2	0.008
67.2	0.217	54	0.180	71.4	0.125	49.2	0.056	69	0.008
81.6	0.222	61.2	0.183	81.6	0.127	57	0.060	72	0.008
94.8	0.227	83.4	0.187	91.2	0.130	61.2	0.061	78.6	0.008
112.8	0.231	99.6	0.188	96.6	0.130	73.8	0.064	82.2	0.008
125.4	0.232	106.2	0.188	105	0.131	85.2	0.065	87.6	0.009
128.4	0.233	115.2	0.188	116.4	0.135	112.2	0.069	103.8	0.009
135	0.235	130.2	0.189	122.4	0.136	121.8	0.069	120.6	0.010
141	0.237	138.6	0.190	130.2	0.138	126.6	0.070	123.6	0.010

Table S. 7. Experimental solubility data for CO_2 in [BzPy][Cl] at different pressures

Time (min)	20 bar χ	Time (min)	15 bar χ	Time (min)	10 bar χ	Time (min)	5 bar χ	Time (min)	1 bar χ
0	0	0	0	0	0	0	0	0	0
1.08	0.025	6	0.051	1.44	0.012	4.68	0.019	3.24	0.002
3.24	0.066	11	0.081	6.12	0.044	7.56	0.025	6.12	0.004
7.56	0.103	15	0.093	9.36	0.056	11.52	0.032	8.64	0.005
9.00	0.109	19	0.106	14.4	0.068	16.2	0.037	11.88	0.006
11.88	0.120	22	0.114	19.44	0.075	19.44	0.040	19.8	0.007
16.92	0.128	25	0.119	25.92	0.079	24.48	0.043	22.32	0.007
27.36	0.137	27.72	0.121	32.76	0.082	30.96	0.043	24.48	0.007
31.68	0.139	32.4	0.123	40.68	0.083	34.56	0.044	27	0.007
36.72	0.141	35.28	0.125	44.64	0.085	38.52	0.044	30.96	0.007
39.96	0.142	41.76	0.125	51.12	0.087	42.48	0.045	32.4	0.008
47.52	0.145	46.08	0.126	54	0.087	48.6	0.045	37.08	0.008
52.2	0.146	51.48	0.127	61.92	0.088	51.12	0.046	38.88	0.008
58.32	0.148	57.96	0.128	64.44	0.088	59.04	0.046	42.84	0.008
65.52	0.149	59.76	0.128	71.28	0.091	61.56	0.046	45	0.008
70.92	0.150	68.04	0.130	75.24	0.092	67.68	0.047	51.84	0.008
77.04	0.151	82.08	0.130	78.84	0.093	71.64	0.047	57.96	0.009
84.24	0.151	82.44	0.130	81.72	0.093	74.16	0.047	68.04	0.009
88.56	0.151	86.4	0.130	83.52	0.093	84.24	0.049	75.24	0.009
91.8	0.151	89.28	0.130			86.04	0.048	84.96	0.009

Table S. 8. Experimental solubility data for CO_2 in [AyPy][Cl] at different pressures

Time (min)	20 bar χ	Time (min)	15 bar χ	Time (min)	10 bar χ	Time (min)	5 bar χ	Time (min)	1 bar χ
0	0	0	0	0	0	0	0	0	0
1.08	0.016	4	0.032	1.44	0.008	1.8	0.002	5.76	0.002
1.8	0.030	8	0.057	5.76	0.032	3.96	0.005	8.64	0.003
3.6	0.051	9	0.064	7.56	0.041	6.48	0.009	10.44	0.004
6.12	0.071	11	0.074	10.44	0.050	8.64	0.012	16.2	0.004
7.56	0.083	14	0.083	13.68	0.057	11.16	0.015	24.12	0.004
12.6	0.102	17	0.089	18.36	0.064	15.48	0.020	26.64	0.004
18.72	0.115	20	0.097	22.68	0.068	18.72	0.025	28.8	0.004
21.24	0.120	23.04	0.102	28.8	0.071	20.52	0.027	31.32	0.005
28.44	0.123	26.28	0.104	35.28	0.073	23.76	0.031	35.28	0.005
36.36	0.128	28.8	0.106	38.16	0.073	27	0.033	36.72	0.005
40.32	0.130	32.4	0.108	42.84	0.075	29.52	0.033	41.4	0.005
48.96	0.133	36.72	0.110	48.96	0.076	34.2	0.036	43.2	0.005
56.88	0.136	50.04	0.112	54.72	0.078	36.72	0.037	47.16	0.005
67.68	0.138	59.76	0.113	57.96	0.078	44.28	0.038	49.32	0.005
75.24	0.139	63.72	0.113	63	0.079	51.12	0.039	52.56	0.005
77.04	0.140	69.12	0.113	69.84	0.081	67.32	0.041	62.28	0.005
81	0.141	78.12	0.113	73.44	0.082	73.08	0.041	72.36	0.006
84.6	0.142	83.16	0.114	78.12	0.083	75.96	0.042	47.16	0.006

Table S. 9. Experimental density values (ρ) for the studied ILs at atmospheric pressure as a function of temperature (T).

T/K	ρ/(g.cm^{-3})					
	[HEA][Ac]	[HEA][Bu]	[HEA][La]	[HEA][As]	[AyHEA][Cl]	[BzHEA][Cl]
298.15	1.18982	1.12571	1.23011	1.05799	1.3001	1.3122
308.15	1.18312	1.11832	1.22122	1.04989	1.2920	1.3028
318.15	1.17447	1.11036	1.21425	1.04259	1.2825	1.2947
328.15	1.16551	1.10276	1.20564	1.03383	1.2744	1.2852
338.15	1.15663	1.09535	1.19676	1.02589	1.2650	1.2771
348.15	1.14976	1.08749	1.18988	1.01777	1.2569	1.2690

Table S. 10. Experimental viscosity values (η) for the studied ILs at atmospheric pressure as a function of temperature (T).

T/K	η/(mPa.s)					
	[HEA][Ac]	[HEA][Bu]	[HEA][La]	[HEA][As]	[AyHEA][Cl]	[BzHEA][Cl]
298.15	470.44	1427.18	858.29	2976.21	1205.40	1314.95
308.15	210.45	586.88	381.46	1239.83	503.91	545.84
318.15	92.25	291.31	169.54	553.89	248.11	271.03
328.15	40.99	143.03	75.35	257.55	117.06	131.08
338.15	18.221	63.57	33.49	114.469	47.81	55.10
348.15	8.098	28.25	14.88	50.875	21.56	24.93

Table S. 11. First-order saturation model fitted parameters

**IL**	q_e	**95% CI**	k	**95% CI**
[HEA][As]	0.5719	0.5581–0.5856	0.0703	0.0612–0.0795
[HEA][La]	0.5570	0.5346–0.5794	0.0542	0.0441–0.0642
[HEA][Bu]	0.5403	0.5276–0.5530	0.0513	0.0468–0.0557
[HEA][Ac]	0.4031	0.4002–0.4059	0.0726	0.0699–0.0753
[BzHEA][Cl]	0.2437	0.2392–0.2483	0.0933	0.0834–0.1032
[AyHEA][Cl]	0.2261	0.2210–0.2312	0.0699	0.0627–0.0771
[BzPy][Cl]	0.1461	0.1434–0.1489	0.1556	0.1391–0.1722
[AyPy][Cl]	0.1356	0.1325–0.1386	0.1158	0.1040–0.1277
